# From Fingerprinting to Advanced Machine Learning: A Systematic Review of Wi-Fi and BLE-Based Indoor Positioning Systems

**DOI:** 10.3390/s25226946

**Published:** 2025-11-13

**Authors:** Sara Martín-Frechina, Esther Dura, Ignacio Miralles, Joaquín Torres-Sospedra

**Affiliations:** 1 Department of Computer Science, ETSE, University of Valencia, Avda. de la Universidad, S/N, 46100 Burjassot, Valencia, Spain; sara.martin-frechina@uv.es (S.M.-F.); esther.dura@uv.es (E.D.); ignacio.miralles@uv.es (I.M.); 2 Valencian Graduate School and Research Network of Artificial Intelligence (VALGRAI), Camí de Vera S/N, Edificio 3Q, 40022 Valencia, Spain

**Keywords:** Indoor Positioning System (IPS), IEEE 802.11 Wireless LAN (Wi-Fi), Bluetooth Low Energy (BLE), Received Signal Strength Indicator (RSSI), Channel State Information (CSI), Round Trip Time (RTT), Angle of Arrival (AoA), Machine Learning (ML), Deep Learning (DL)

## Abstract

The Indoor Positioning System (IPS) is used to locate devices and people in smart environments. In recent years, position determination methods have evolved from simple Received Signal Strength Indicator (RSSI) measurements to more advanced approaches such as Channel State Information (CSI), Round Trip Time (RTT), and Angle of Arrival (AoA), increasingly combined with Machine Learning (ML). This article presents a systematic review of the literature on ML-based IPS using IEEE 802.11 Wireless LAN (Wi-Fi) and Bluetooth Low Energy (BLE), including studies published between 2020 and 2024 under the Preferred Reporting Items for Systematic Reviews and Meta-Analyse (PRISMA) methodology. This study examines the techniques used to collect measurements and the ML models used, and discusses the growing use of Deep Learning (DL) approaches. This review identifies some challenges that remain for the implementation of these systems, such as environmental variability, device heterogeneity, and the need for calibration. Future research should expand ML applications to RTT and AoA, explore hybrid multimetric systems, and design lightweight, adaptive DL models. Advances in wireless standards and emerging technologies are also expected to further enhance accuracy and scalability in next-generation IPS.

## 1. Introduction

In recent years, the Indoor Positioning System (IPS) has attracted considerable attention due to its numerous applications, including asset tracking, emergency response, smart environments, and navigation in large buildings. However, the lack of Line-of-Sight (LOS) in complex indoor environments, multipath propagation, and signal degradation make indoor localization a difficult challenge in contrast to outdoor positioning, which is consistently supported by satellite-based systems such as Global Positioning System (GPS).

The development of indoor positioning systems began to take shape with the introduction of RADAR [1] in 2000, one of the first Radio Frequency (RF)-based location systems that introduced the K-Nearest Neighbour (KNN) and Wi-Fi Received Signal Strength Indicator (RSSI) fingerprinting approach. This approach demonstrated the feasibility of indoor positioning using signal strength patterns. Years later, with the advent of Bluetooth Low Energy (BLE) [2] in around 2015, similar fingerprinting strategies were adapted to BLE beacons, taking advantage of the technology’s low power consumption and its compatibility with mobile devices. For several years, fingerprinting using RSSI and KNN has been the dominant approach in the field due to its simplicity and low deployment cost. RSSI-based identification combined with simple algorithms like KNN proved effective thanks to the low dimensionality of the measurements and their ease of implementation. However, as more complex signal measurements (CSI, RTT, AoA) became available, the need for more advanced ML models became evident, since traditional algorithms are not well-suited for capturing their complexity.

However, in recent years, this approach has declined in prominence due to its limitations in dynamic and multipath environments. Advanced signal measurements such as Channel State Information (CSI), Round Trip Time (RTT), and Angle of Arrival (AoA) have gained increasing attention, allowing for more accurate and robust localization. For example, CSI-based DL models have achieved sub-metre accuracy by leveraging the detailed multipath information provided by CSI to distinguish locations in complex indoor environments [3]. Similarly, RTT-based approaches that leverage the IEEE 802.11mc protocol offer increased robustness in complex indoor settings by using precise timing measurements to estimate distances, thereby improving accuracy in multipath environments [4]. BLE 5.1 AoA-based systems have also demonstrated significant improvements in indoor localization, leveraging direction-finding techniques to achieve precise angle estimation and sub-metre accuracy [5]. These advances, combined with the rise of more sophisticated ML techniques, have led to a paradigm shift in the design of indoor positioning systems.

Among the various technologies explored for indoor localization, BLE and IEEE 802.11 Wireless LAN (Wi-Fi) have emerged as two of the most prominent options, primarily due to their widespread availability in consumer devices, cost-effectiveness, and energy efficiency. These technologies provide signal measurements such as the RSSI, CSI, RTT, Direction of Arrival (DoA) and AoA, which can be exploited to estimate the position of a device within an indoor space.

With increasing demand for indoor positioning systems in applications such as asset tracking, navigation, and smart environments, studies that synthesize advances in technologies like BLE and Wi-Fi are critical. In parallel, the rapid evolution of ML techniques has opened new possibilities for enhancing the performance of IPS. By leveraging ML algorithms, it is possible to model complex signal behaviors, mitigate measurement noise, and improve positioning accuracy in dynamic and Non Line-of-Sight (NLOS) environments. Therefore, the integration of ML with BLE and Wi-Fi-based positioning has emerged as a major research focus over the past five years.

**Figure 1:**
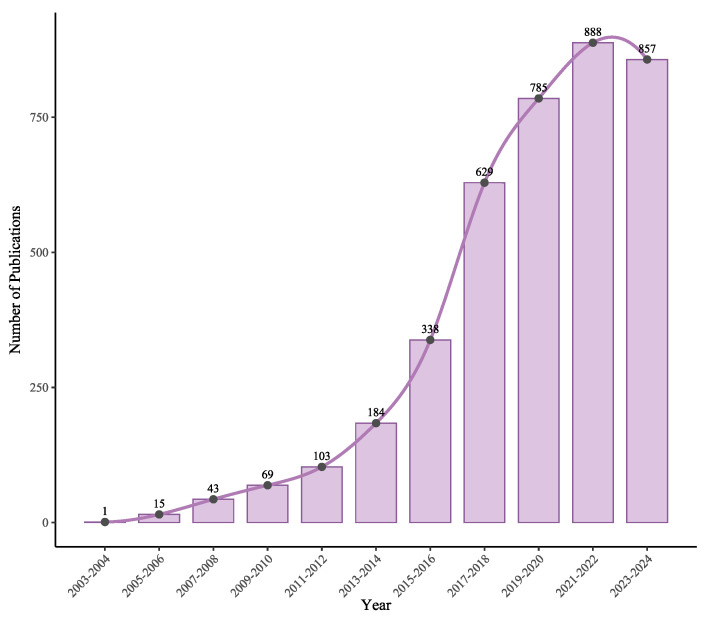
Number of publications per two-year interval in the Scopus database on BLE/Wi-Fi Indoor Positioning using Machine Learning. Data were retrieved using the following query: (TITLE-ABS-KEY (“Indoor Position*” OR “Indoor Track*” OR “Indoor Locati*” OR “Indoor Locali*” OR “Indoor Navigat*”) AND TITLE-ABS-KEY (“BLE” OR “Bluetooth Low Energy” OR “Wi-Fi” OR “WLAN” OR “Wifi” OR “802.11”) AND ALL (“Machine Learning” OR “ML” OR “Deep Learning” OR “DL” OR “Neural Networks” OR “NN” OR “KNN” OR “K-nearest” OR “SVM” OR “Support Vector Machine”)).

Despite the growing body of literature in this field (see Figure 1), there is currently no comprehensive survey or review that encompasses the most recent developments. Therefore, a systematic analysis and synthesis of recent research are necessary to capture the current state of the art, identify dominant techniques and emerging trends, and reveal existing knowledge gaps. This Systematic Literature Review (SLR) aims to address this need by providing a comprehensive overview of ML-based indoor positioning approaches using BLE and Wi-Fi technologies from 2020 to 2024, following the Preferred Reporting Items for Systematic Reviews and Meta-Analyse (PRISMA) [6] methodology.

The objective of this study is to examine the evolution of Wi-Fi- and Bluetooth Low Energy (BLE)-based Indoor Positioning System (IPS) in terms of the signal measurements employed—namely, Received Signal Strength Indicator (RSSI), Channel State Information (CSI), Round Trip Time (RTT), and Angle of Arrival (AoA)—and the Machine Learning (ML) methods used to process these measurements. The main contribution of this work is to provide a systematic and up-to-date review of ML-based indoor positioning solutions that rely on Wi-Fi and BLE as the primary technologies, with a particular focus on recent advances in positioning techniques leveraging AoA, CSI, and RTT. The results of the analysis indicate that ML-based IPS have progressively shifted from traditional algorithms—such as K-Nearest Neighbour (KNN), Supported Vector Machine (SVM), and Decision Tree (DT)—toward Deep Learning (DL) architectures, including Convolutional Neural Networks (CNNs), Deep Neural Networks (DNNs), and Long Short-Term Memory (LSTM) models. A clear trend has emerged toward exploiting advanced signal measurements (CSI, RTT, and AoA) to achieve sub-meter accuracy. In particular, BLE AoA and Wi-Fi RTT have demonstrated significant improvements compared to conventional RSSI-based approaches, while CSI-based methods, though less extensively studied, have shown promising results when combined with neural networks. These findings highlight both the maturity of RSSI-based research and the emerging potential of next-generation measurements for achieving more reliable and scalable indoor localization.

The rest of this document is organized as follows. Section 2 introduces the related work in terms of previous surveys/analysis, providing the context for this updated systematic review. Section 3 describes the systematic literature review process, including the search strategy, inclusion and exclusion criteria, and classification framework based on the PRISMA methodology [6]. Section 4 presents the main findings in response to the defined research questions, covering the use of ML techniques in RSSI, CSI, RTT, and AoA-based positioning systems. Section 5 discusses the main findings, synthesizes answers to research questions, and explores ongoing challenges and open issues in the field. Finally, Section 6 summarizes key findings, draws conclusions, and discusses potential directions for future research directions.

## 2. Related Work

Over the last decade, numerous surveys and review articles have addressed the growing field of IPS. These studies have explored various technologies such as Wi-Fi, BLE, Radio Frequency Identification (RFID), Ultra-Wideband (UWB), and Visible Light Communication (VLC), alongside signal metrics like RSSI. Earlier works primaly introduced classical Machine Learning (ML) approaches for location estimation, while more recent works incorporate Deep Learning (DL) models to achieve enhanced positioning accuracy and robustness.

It is important to point out that Deep Learning (DL) is a subfield of Machine Learning (ML). Unlike classic ML methods, which often rely on manually created features and basic models, DL approaches are characterized by the use of deeper neural network architectures capable of automatically automatically extracting complex features from raw signal data.

In the following, we provide a chronological analysis of the most relevant surveys in this area. For each study, we highlight their main contributions as well as their weaknesses, especially focusing on the areas that remain unexplored. This comparative perspective allows us to contextualize the contribution of the present study in relation to the available literature. Table 1 summarizes these surveys, providing an overview of their scope, focus, and limitations.

An early study by Ding et al. [7] focuses on RSSI-based fingerprinting in WLAN environments, addressing challenges, such as RSSI variability, and proposing classical solutions like probabilistic modeling and sensor fusion. Similarly, Sakpere et al. [8] provide a review of positioning technologies, including infrared, ultrasound, RFID, and visible light, comparing their accuracy, cost, and privacy. Despite their valuable contributions, both works were published before the emergence of Wi-Fi RTT, BLE AoA, and DL. Consequently, these aspects, which form the focus of this study, remain unexplored in their analyses.

A survey of non-GPS positioning by Tariq et al. [9] examines BLE and Wi-Fi and hybrid approaches. While informative, the review is limited to traditional ML models and omits recent signal metrics such as AoA, CSI, and advances DL methods like CNNs or transformers. Similarly, Zafari et al. [10] presents a comprehensive overview of IPS technologies and their applications in Internet of Things (IoT) environments. Although they address Wi-Fi and BLE, the analysis remains focused on deterministic and classical ML models, with no coverage of DL architectures. SimilarlyObeidat et al. [11] extend the scope to include UWB and evaluate multiple factors such as accuracy, coverage, and energy efficiency. However, their work is still confined to traditional algorithms such as KNN and overlooks key advances in signal metrics and DL. These surveys, while valuable for understanding the early stages of ML in indoor localization, do not capture the paradigm shift introduced by recent technologies like Wi-Fi RTT, BLE 5.1 AoA, or DL-based approaches, dimensions that our review integrates in depth.

Singh et al. [12] focus their analysis on Wi-Fi RSSI fingerprinting with ML, providing useful information on preprocessing and the use of neural networks. However, the review does not explore emerging metrics, like RTT or AoA, and does not consider recent DL approaches or a cross-technology analysis. In contrast, the 3D indoor localization survey by Sesyuk et al. [13] addresses Wi-Fi and BLE, explicitly reporting results with RSSI-based methods for both. Nevertheless, it does not analyze Wi-Fi RTT, BLE AoA, or CSI, and it offers only a limited discussion of recent DL approaches.

Feng et al. [14] present a review of Wi-Fi-based indoor positioning that emphasizes the use of DL approaches for processing CSI, with applications in Human Activity Recognition(HAR). Although the study highlights the potential of DL, it omits BLE AoA, and Wi-Fi RTT. Simelane et al. [15] provide a broad overview of RF-based IPS, covering Wi-Fi and BLE, focusing on techniques such as fingerprinting and trilateration, with applications in navigation. While their review includes a discussion on ML, it does not specifically address DL methods, an area that this study explores in greater depth.

More recent surveys have begun to explore modern approaches, yet important gaps remain. For instance, Dai et al. [16] classify Wi-Fi-based IPS solutions using RSSI and Fine Timing Measurement (FTM) ranging, incorporating some DL models. However, their reviews omit BLE AoA and restrict their scope exclusively to Wi-Fi systems. Similarly, Park et al. [17] evaluate ML models like XGBoost and Random Forest (RF) for Wi-Fi RSSI-based localization, but they do not address advanced signal metrics or DL, nor include BLE-based approaches.

Bastiaens et al. [18] present a review on Visible Light Positioning (VLP), which is considered as one of the next-generation indoor positioning technologies. The study offers a detailed description of the fundamentals, components, and architectures of VLP systems, analyzing both hardware aspects and localization algorithms. Although this work represents a valuable contribution, its analysis focuses on the technical aspects of visible light and does not address Wi-Fi and BLE technologies or the incorporation of ML, which constitutes the main focus of this review.

Morgan [19] also study BLE-based systems, discussing ML techniques and challenges such as multipath. However, their work does not address DL models.

Singh et al. [20] conduct a systematic review of IPS, proposing a taxonomy that organizes technologies, techniques, and algorithms. They also discuss key issues related to security, privacy, and practical challenges for adoption in real-world settings. However, although the review acknowledges the role of ML, it does not explore the emerging metrics that constitute the focus of this research.

Lin et al. [21] presents a review focused on Wi-Fi-based indoor localization systems, with particular emphasis on DL models. The article classifies architectures, such as CNNs, Recurrent Neural Networks (RNNs), autoencoders, and hybrid models, and also analyzes data preprocessing strategies and noise reduction techniques applied to RSSI and CSI. Although it stands as one of the most comprehensive contributions in the field of DL for Wi-Fi, it does not address BLE or emerging measurement approaches.

Some recent work focuses specifically on BLE. For instance, Shi and Gong [22] investigate BLE-based localization, discussing ML techniques and applications for indoor navigation. However, their analysis does not consider Wi-Fi RTT, DL approaches, or comparative evaluations across technologies.

Finally, Ahmad et al. [23] presents one of the few reviews integrating DL models for HAR using CSI. While valuable, their review omits BLE AoA and Wi-Fi RTT.

As the chronological analysis and the summary in Table 1 demonstrate, previous reviews have provided valuable but fragmented perspectives. They tend to focus on a single technology (Wi-Fi or BLE), a single signal metric (RSSI or CSI), or a limited time frame that misses the recent explosion in advanced techniques. To our knowledge, no prior work has conducted a systematic review that simultaneously synthesizes the evolution of both Wi-Fi and BLE-based systems while covering the full spectrum of modern signal metrics, CSI, RTT, and AoA, alongside the rise of DL.

This study fills this critical gap. Our unique contribution is this holistic and up-to-date synthesis, covering the years 2020 to 2024. By analyzing these technologies and metrics in parallel, we provide a comprehensive understanding of the current state of the art, identify cross-technology trends, and highlight the specific research frontiers for the next generation of indoor positioning systems.

While this review focuses on Wi-Fi and BLE technologies due to their ubiquity and cost-effectiveness, it is important to position them within the broader landscape of indoor positioning solutions. Other prominent technologies offer different trade-offs. For instance, UWB systems are renowned for their centimeter-level accuracy but require the deployment of dedicated infrastructure, increasing costs and complexity. Similarly, VLC offers high-precision positioning and is immune to radio frequency interference; however, its reliance on LOS between transmitters (LED lights) and receivers limits its applicability in cluttered environments.

Furthermore, Inertial Measurement Units (IMUs), integrated into most smartphones, provide a powerful means for dead-reckoning but suffer from cumulative drift error over time. Consequently, IMUs are rarely used in isolation and are most effective when fused with RFID-based technologies like Wi-Fi or BLE to periodically correct their position estimates. Therefore, the focus of this review on Wi-Fi and BLE reflects their unique balance of acceptable accuracy, widespread infrastructure availability, and low deployment cost, making them a dominant area of research for scalable, real-world applications.

## 3. Research Methodology

This section outlines the methodology and process followed to conduct the systematic review on IPSs based on Bluetooth Low Energy (BLE) and Wi-Fi technologies. The Preferred Reporting Items for Systematic Reviews and Meta-Analyse (PRISMA) methodology has been selected for its clarity and reproducibility, enabling other researchers to replicate the process and obtain comparable results. It aims to comprehensively analyze the existing literature, identify current techniques and trends, and uncover potential research gaps within the domain of indoor localization.

### 3.1. PRISMA Workflow

To guide this review, we adhered to the PRISMA guidelines [6] in Supplementary Material. These guidelines provide a structured approach for conducting systematic reviews and include a twenty-seven-item checklist along with a flow diagram, divided into four phases: identification, screening, eligibility, and inclusion.

The review protocol begins with the formulation of research questions that define the scope and objectives of the study. Based on these questions, a set of inclusion and exclusion criteria were established to ensure that only relevant and high-quality studies were selected for analysis.

The study selection process involved defining relevant search queries tailored to the topic of indoor positioning systems and the scientific dataset. The initial search results were combined, and duplicate records were removed. The remaining articles were then screened using the predefined inclusion and exclusion criteria to identify the final set of relevant studies.

Once the final set of articles was identified, each study was carefully analyzed, categorized, and mapped according to the established research questions. This process involved extracting key features, including the positioning techniques employed and the evaluation metrics used.

The following subsections provide a detailed description of the research questions (Section 3.2), the inclusion and exclusion criteria (Section 3.3), the query (Section 3.4), and an overview of the results in our case study (Section 3.5).

### 3.2. Research Questions

Defining research questions is a fundamental step in any systematic review, as it establishes the main objectives of the analysis. In this work, the review focuses on indoor localization using BLE and Wi-Fi technologies.

To guide the process, a Main Research Question (MRQ) was defined which acts as the central axis of the research.

**MRQ:** How have ML-based methods applied to Wi-Fi or BLE-based indoor positioning systems evolved over the last five years?

As this main question is generic, it is divided into the following specific Research Questions (RQs):

**RQ1:** What ML methods have been used in positioning systems based on individual Wi-Fi and BLE using RSSI?
**RQ2:** What ML methods are currently used in Wi-Fi CSI, Wi-Fi RTT, and BLE AoA for indoor positioning?
**RQ3:** What have been the main accuracy improvements in moving from RSSI-based techniques to techniques such as CSI, RTT, and AoA in Wi-Fi and BLE?
**RQ4:** What are the main challenges and limitations in Wi-Fi and BLE-based positioning systems?
**RQ5:** What are the least explored areas in the application of ML algorithms in these indoor positioning technologies?

### 3.3. Selection Criteria

Carefully defined Inclusion Criteria (IC) and Exclusion Criteria (EC) were established to filter the records, ensuring that only relevant high-quality studies addressing IPS based on BLE, Wi-Fi, and ML were included for further analysis.

**IC1:** Any full primary research article written in English.
**IC2:** Any article published in international peer-reviewed journals or international peer-reviewed conference proceedings.
**IC3:** Studies that analyze algorithms applied to indoor positioning systems based on Wi-Fi or BLE.
**EC1:** Articles that do not employ RSSI, CSI, RTT, AoA or DoA as primary positioning techniques or primarily rely on other technologies or studies that use hybrid positioning approaches.
**EC2:** Articles that do not use ML in the positioning process.
**EC3:** Articles that do not focus exclusively on indoor positioning, addressing other topics such as posture recognition, movement direction detection, or activity classification.
**EC4:** Articles that do not include real-world validation, relying solely on simulations without experimental verification in real environments.
**EC5:** Articles lacking sufficient data for reproducibility due to missing critical experimental details, such as surface area, localization error, or other essential parameters.

By applying these criteria, the review ensures that only relevant and high-quality studies are included, addressing the research objectives.

### 3.4. Search Queries

The core of the generic search query was designed to identify studies related to indoor positioning systems based on BLE and Wi-Fi technologies. For this purpose, two well-established scientific databases—Scopus and Web of Science—were selected, as they index the majority of venues relevant to the positioning domain. The generic query was subsequently adapted to the syntax of each database. The specific search strings used, along with the number of retrieved documents, are summarized in Table 2.

Both queries returned a comprehensive list of works from 2020 to 2024, including journal papers, conference papers, and other types of publications. As shown in the table, 1081 documents were retrieved from Scopus, while 537 documents were retrieved from Web of Science. Although many of the recovered records were relevant to the research topic, some did not meet the inclusion criteria established for this review, as detailed in the following sections.

### 3.5. PRISMA Process Overview and Results

**Figure 2:**
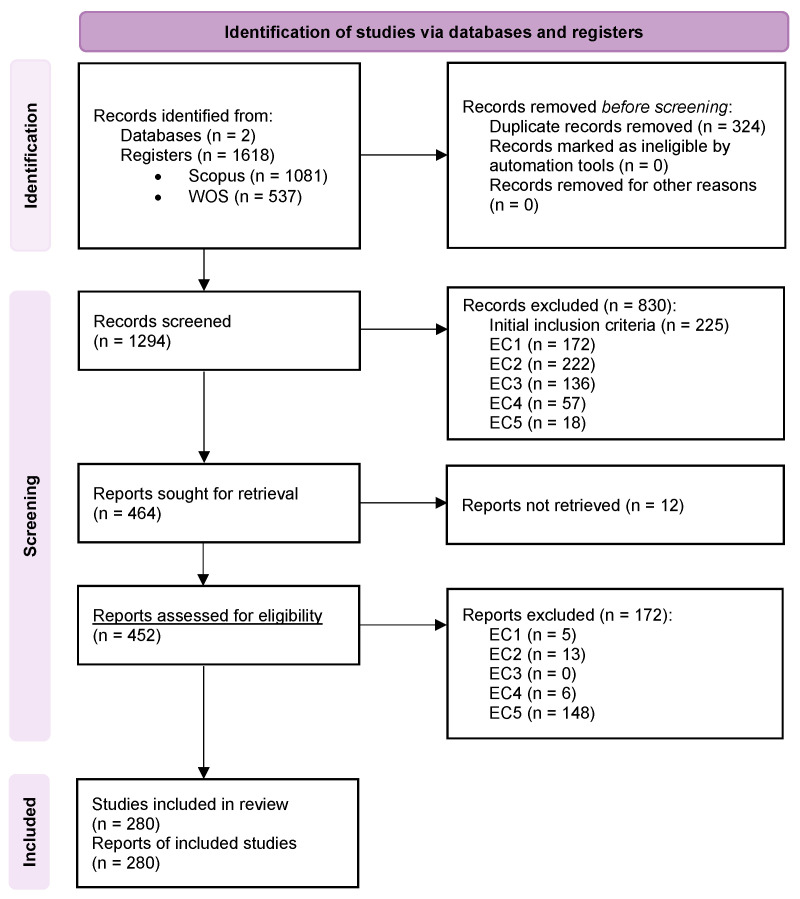
PRISMA flow diagram.

Figure 2 illustrates the flow diagram of our systematic literature review, following the PRISMA 2020 statement. The process began with the identification of records from two widely recognized scientific databases. As detailed in Table 2, our search queries retrieved a total of 1618 records published between 2020 and 2024: 1081 from Scopus and 537 from Web of Science.

In the first step of the screening phase, 324 duplicate records were identified and removed using the reference management software Zotero to ensure that each study was considered only once. This left 1294 unique articles for title and abstract screening. During this stage, 830 records were excluded because they did not meet the predefined inclusion criteria (wrong topic, incorrect publication type) or met one of the exclusion criteria (a detailed breakdown is provided in Figure 2).

This left 464 records for full-text retrieval. We were unable to retrieve 12 articles, resulting in 452 reports evaluated for eligibility. In the final stage, after a thorough evaluation of the full text, another 172 studies were excluded because they met one of the exclusion criteria.

This rigorous screening process resulted in a final set of 280 studies that were included in our comprehensive analysis.

### 3.6. Quality Assessment and Risk of Bias

To ensure the methodological rigor of this review and assess the reliability of the included studies, a quality assessment was performed on all articles that passed the full-text screening. While formal risk-of-bias tools, such as those used in clinical reviews, are not standard practice in this engineering domain, we adopted a qualitative quality checklist based on established systematic review principles. Each included study was evaluated against the following criteria:

- Clarity of Objectives and Methodology: The study must clearly state its research questions, objectives, and the methodology used to address them.
- Completeness of Experimental Setup: The description of the experimental environment (area size, layout, number of Access Points (APs)/beacons) and hardware (device models) must be sufficiently detailed.
- Reproducibility of Results: The performance metrics used (mean error, RMSE) must be clearly defined, and the evaluation process should be transparent enough to allow for conceptual replication.
- Presence of a Baseline for Comparison: The study should compare its proposed method against at least one established baseline or state-of-the-art approach to contextualize its performance.

Studies that failed to meet these fundamental quality standards were excluded, as noted in the PRISMA flow diagram (Figure 2). Furthermore, the primary risk of bias identified across the body of literature is related to the generalizability of findings. Many studies are conducted in controlled, small-scale laboratory or office environments, which may lead to an optimistic estimation of performance that is not replicable in larger, more dynamic real-world scenarios such as shopping malls or airports. By explicitly considering these quality criteria and acknowledging this potential bias, our review aims to provide a balanced and critical synthesis of the field.

## 4. Results

This section analyzes insights from 280 studies to address research questions in Section 3.2. It examines the evolution of ML-based methods in indoor BLE and Wi-Fi positioning systems, focusing on signal metrics, algorithmic performance, and practical challenges.

### 4.1. Characterization and Analysis of Included Literature

From the initial records identified through the PRISMA process (see Figure 2), 280 studies met the criteria for our systematic literature review on BLE and Wi-Fi-based Indoor Positioning System (IPS) using Machine Learning (ML), which forms the basis of our investigation.

**Figure 3:**
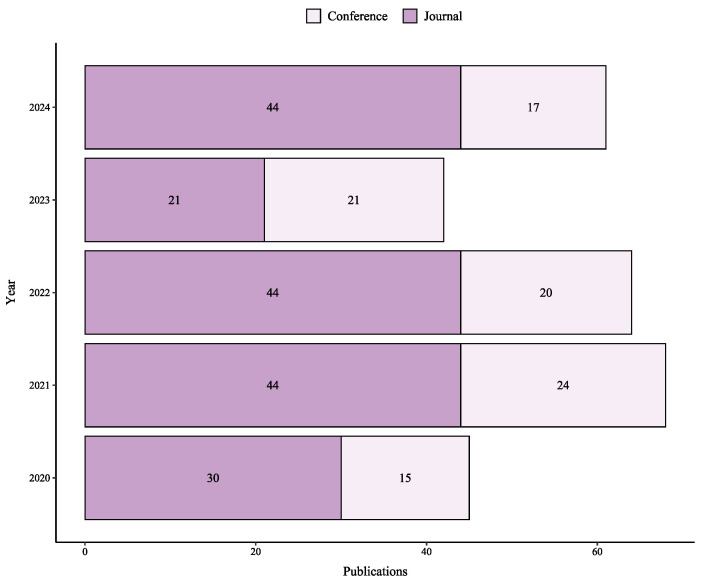
Distribution of selected studies by publication type and year.

Figure 3 presents the publication timeline of these studies from 2020 to 2024, breaking down the included works into 97 conference papers and 183 journal articles. The significant proportion of publications in peer-reviewed journals underscores the maturity of the research field. In our review, both publication types were assessed against the same inclusion and quality criteria outlined in Section 3.3, ensuring that all included studies met a consistent standard of scientific rigor.

Research activity peaked in 2021 with 68 publications and declined to 42 in 2023. This five-year fluctuation indicates varying levels of interest in the field, potentially influenced by post-pandemic recovery challenges or a shift toward related application areas.

**Figure 4:**
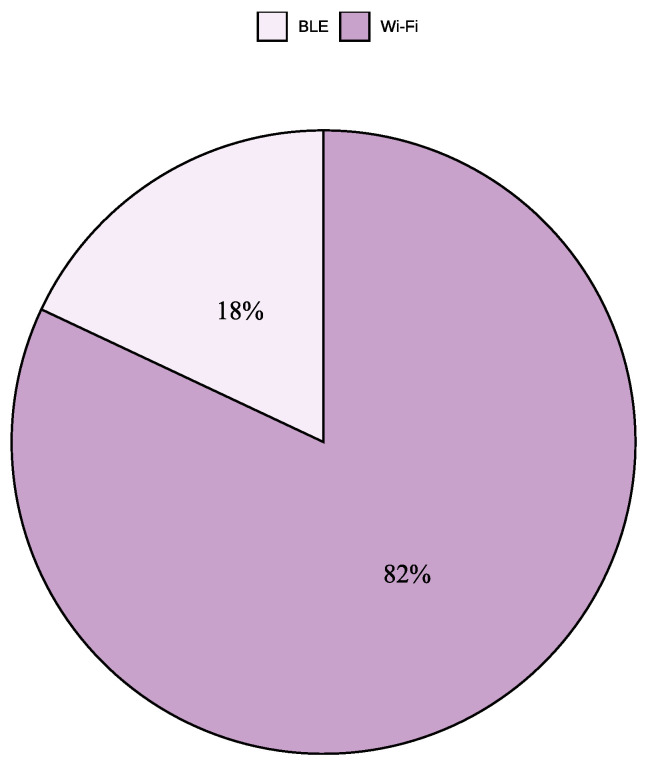
Percentage of Studies by Technology.

**Figure 5:**
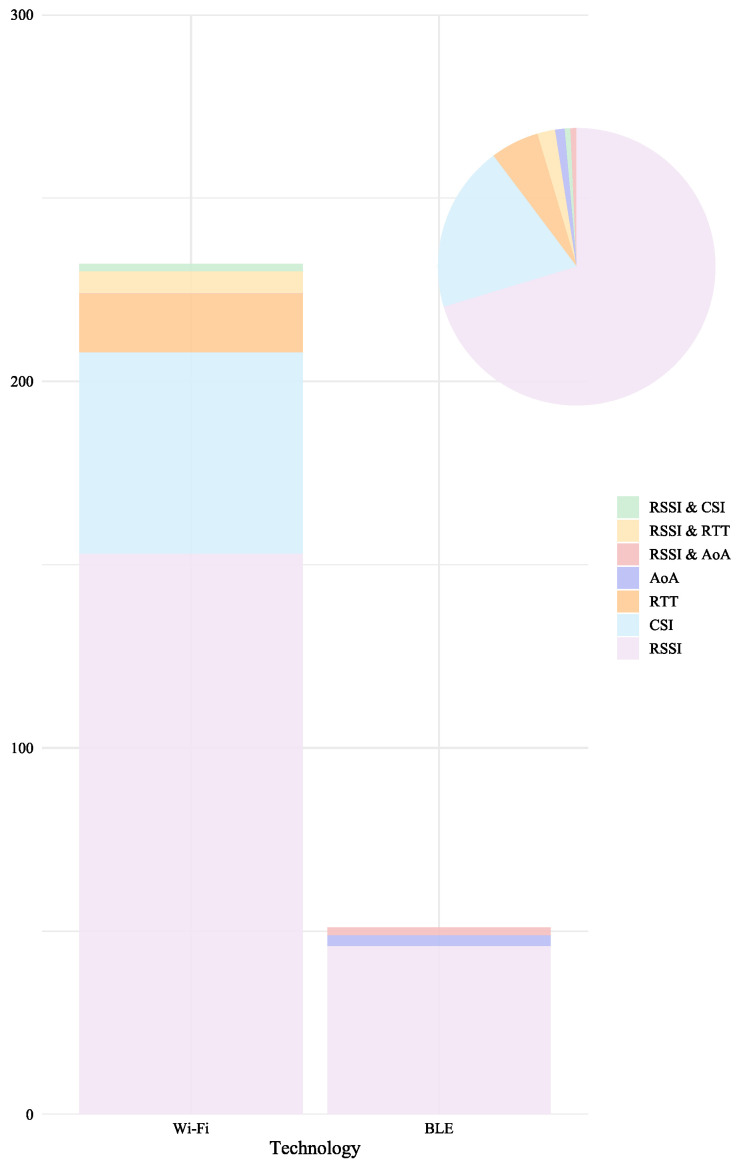
Percentage of studies by signal metric.

To characterize the technological focus of the selected literature, we analyzed the distribution of the 280 studies included in this review. As shown in Figure 4, 82% of the studies employed Wi-Fi, while 18% focused on BLE, highlighting the dominant role of Wi-Fi in IPS research. Furthermore, Figure 5 illustrates that 70% of the studies (196 out of 280) utilized RSSI, 19.6% (55 out of 280) relied on CSI, 5.7% (16 out of 280) adopted RTT, and 1.1% (3 out of 280) incorporated AoA. In addition, 2.1% (6 out of 280) combined RSSI and RTT, 0.7% (2 out of 280) combined RSSI and CSI, and 0.7% (2 out of 280) combined RSSI and AoA, confirming the prevalence of RSSI as the most widely used signal metric.

The systematically compiled data encompass the utilization of signal metrics (RSSI, CSI, RTT, and AoA); the application of ML algorithms such as KNN, CNNs, and LSTM; testing environments; reported performance indicators; and key challenges or advancements noted by the authors. These findings, derived from studies published between 2020 and 2024, address the research questions outlined in Section 3.2 and are further detailed in Section 4. Together, the technological and signal metric distributions provide a foundational overview of the current state of the field.

### 4.2. Machine Learning Methods Employed in Wi-Fi and BLE-Based Indoor Positioning Systems Using RSSI (RQ1)

To address RQ1, we analyzed Machine Learning (ML) methods applied to an RSSI-based Indoor Positioning System (IPS) using Wi-Fi and Bluetooth Low Energy (BLE) technologies. RSSI, valued for its simplicity and widespread availability, serves as a key input for IPS, with a significant focus on Wi-Fi and BLE implementations between 2020 and 2024.

#### 4.2.1. Wi-Fi RSSI-Based Indoor Positioning Systems

The analysis of Wi-Fi-based systems revealed a wide variety of ML approaches, summarized and illustrated in Table 3.

Table 3 summarizes the different ML algorithms identified in the selected studies, applied to indoor positioning systems based exclusively on Wi-Fi RSSI. A total of 23 different types of methods were recorded, classified according to the stage of the process in which they are applied (positioning, pre-processing, aggregation, etc.), the number of appearances in the studies, and their bibliographic references. The “occurrences” column represents the number of studies in which each ML method was applied, providing a measure of the prevalence of different approaches within the Wi-Fi RSSI-based indoor positioning literature.

The most commonly applied family of methods was the KNN-based algorithms, which appeared in a total of 54 studies. This group includes 31 occurrences of standard KNN [36,37,38,39,40,41,42,43,44,45,46,47,48,49,50,51,52,53,54,55,56,57,58,59,60,61,62,63,64,65,66], 17 of its weighted variant Weighted K-Nearest Neighbour (WKNN) [67,68,69,70,71,72,73,74,75,76,77,78,79,80,81,82,83], and 6 of Improved K-Nearest Neighbour (IKNN) [84,85,86,87,88,89], confirming its predominant role as a baseline positioning technique in fingerprinting systems.

It is followed by other classical supervised learning methods such as Random Forest (RF) [40,41,46,48,52,55,57,61,62,82,107,108,109,110,111,112,113,114,115,116,117] and Supported Vector Machine (SVM) [40,52,57,59,61,62,116,127,143,144,145,146], as well as other approaches including XGBoost [40,74,107,108,144,156,157], Decision Tree (DT) [41,52,62,111], and Naive Bayes (NB) [55], although these appear less frequently.

There is also a strong presence of DL methods, particularly in recent years. Convolutional Neural Networks (CNNs) were identified in 20 studies [25,27,29,31,33,72,90,91,92,93,94,95,96,97,99,101,102,103,104,106], mainly applied to spatial feature extraction from RSSI fingerprints. LSTM [32,35,53,118,119,120,121,122,123,124,125,126,127,128,129,130], Deep Neural Networks (DNNs) [24,26,34,116,131,132,133,134,135,137,138,139,141,142], and Multilayer Perceptrons (MLPs) [28,59,62,123,134,136,140,141,147,148,149,150,151,152] were also commonly used, suggesting a growing trend toward sequence modeling and the use of complex architectures capable of learning abstract representations from RSSI data.

In the preprocessing stage, several studies have explored alternatives to standard CNNs and DNNs, including autoencoders [24,25,26,27,28,29,30,31,32,33,34,35], which were frequently used for dimensionality reduction or noise removal. In the aggregation stage, clustering methods such as K-means or DBSCAN [44,45,49,64,65,89,106,119,124,158,163] were identified, along with ensemble learning techniques [66,142,150,151,152,153,154,155] that combine multiple models to improve robustness and accuracy.

Furthermore, although less frequent, more recent and complex approaches such as Transformer-based methods [100,173,174,175,176] and domain adaptation or adversarial networks (DANN/GANs) [30,138,168,169,170,171] were also identified, indicating an increasing interest in advanced architectures.

In summary, most of the algorithms are applied directly to the positioning stage of the system, with fewer focused on earlier phases such as pre-processing or result aggregation. This reflects the current research trend toward developing intelligent algorithms that can handle the high variability and uncertainty of RSSI signals.

#### 4.2.2. BLE RSSI-Based Indoor Positioning Systems

The analysis of BLE-based systems revealed a more concentrated set of ML approaches, as summarized and illustrated in Table 4. This table lists the 22 distinct ML algorithm families identified in the selected studies that focus exclusively on BLE RSSI. Each method is classified by its role in the pipeline (positioning, pre-processing, aggregation), the number of appearances, and the relevant references. The “occurrences” column represents the number of studies in which each ML method was applied, providing a measure of the prevalence of different approaches within the BLE RSSI-based indoor positioning literature.

The family most frequently used was KNN-based algorithms, recorded in 22 studies, comprising 18 standard KNN [63,188,189,190,191,192,193,194,195,196,197,198,199,200,201,202,203,204], 7 WKNN [186,188,193,194,204,205,206], and 1 IKNN [207]. This prevalence confirms the enduring popularity of KNN for BLE fingerprinting, attributed to its conceptual simplicity, minimal computational requirements, and reliable performance in low-power environments.

DL techniques, though less widespread, were also present. Convolutional Neural Networks (CNNs) [186,188,194,208,209,210] and other feed-forward neural variants [185,196,211,212,213,214] were each used in six studies, predominantly for positioning tasks involving spatial RSSI pattern extraction. Multilayer Perceptrons (MLPs) [189,203,215,216,217] and Random Forest (RF) [191,199,201,203,207] appeared in five studies each, also focused on positioning. Regression-oriented methods such as Support Vector Regression (SVR) [189,196,218,219] were employed in four studies, followed by Supported Vector Machines (SVMs) [200,203,218], Deep Neural Networks (DNNs) [186,192,220,221], Autoencoders [185,186,187], and Gaussian Process Regression (GPR) [206,219,222], with three appearances each.

Autoencoders were occasionally used during the pre-processing stage, aimed at denoising or dimensionality reduction of RSSI data. For the aggregation stage, K-means clustering [229] was observed in a small number of works, typically to group similar signal patterns or locations before applying a classifier.

Less frequent methods included Feed-Forward Neural Networks (FFNNs) [187,208] and Naive Bayes (NB) [194,199], along with isolated appearances of more specialized or hybrid approaches such as Trilateration [189,223], XGBoost [201], Boosted/Bagged Trees [222], Decision Tree (DT) [200], K-Means [229], Hidden Markov Models (HMMs) [192], Particle Swarm Optimization (PSO) [224], Principal Component Analysis (PCA) [225], Long Short-Term Memory (LSTM) [130], and Transformer-based models [221] (one study each). Additionally, an “Other Methods” [184,219,226,227,228] category grouped five unique techniques that did not fit the standard taxonomies.

In summary, most algorithms are applied during the positioning phase of BLE-based systems, with comparatively few dedicated to preprocessing or result aggregation. This pattern reflects a continuing trend in BLE research toward enhancing positioning accuracy through lightweight and hybrid ML approaches that effectively adapt to the inherent variability of BLE RSSI signals.

#### 4.2.3. Architectural Analysis of CNN and DNN Models Used in Wi-Fi-Based Systems

**Figure 6:**
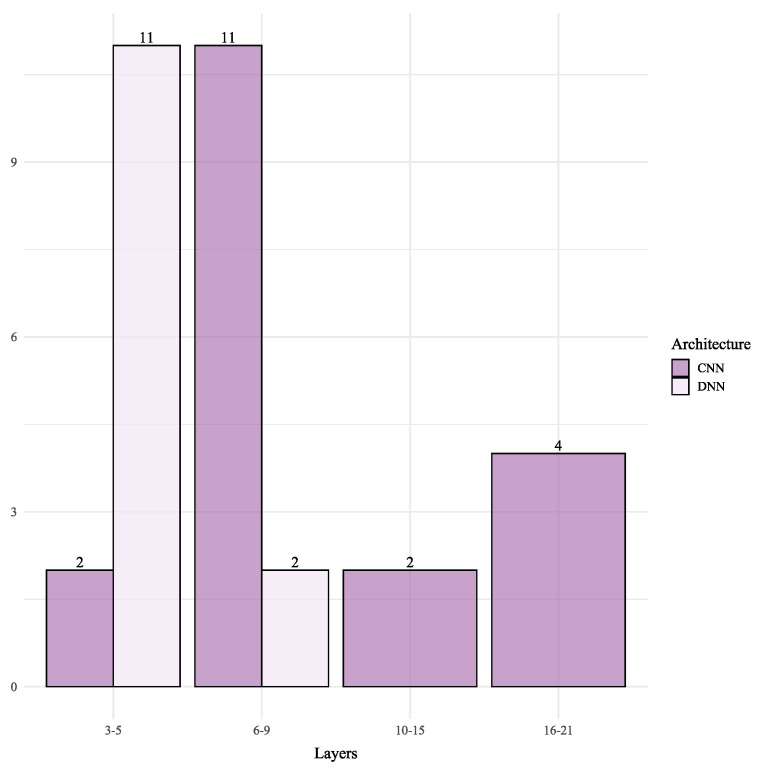
Distribution of CNN and DNN depth in Wi-Fi-based indoor positioning studies.

The architectural design of DL models for Wi-Fi RSSI, detailed in Table 5, reveals a complex interplay between modeling approach, dataset nature, and the trade-off between accuracy and scale. The distribution analysis (Figure 6) shows that Wi-Fi architectures are generally deep, with CNNs often ranging from 7 to 21 layers, while DNNs remain shallower (3–8 layers).

A dominant pattern emerging from the data is that impressive sub-meter accuracy is almost exclusively achieved in highly controlled, small-scale environments. For instance, a 1D-CNN [92] achieved a remarkable 0.058 m error in a small 37.6 m^2^ space, and a regression-focused DL model [132] reported 0.888 m accuracy in a 26 m^2^ office. However, when these architectures are applied to large-scale, real-world public datasets like UJIIndoorLoc, the error increases significantly. For example, a CDAE-CNN hybrid [27] which achieved a 1.05 m error in a small testbed saw its error grow to 12.4 m on the UJIIndoorLoc dataset. Similarly, a DAE-based DNN [26] reported a 6.01 m error on the same large-scale dataset. This clearly indicates that performance metrics cannot be divorced from their environmental context; high accuracy in a lab does not guarantee success in a large, dynamic space.

Another key insight is the effectiveness of hybrid architectures that combine different models or include explicit preprocessing stages. These designs often yield a better balance of accuracy and robustness in challenging environments. For example, the DCCA framework that fuses RSSI with RTT data [141] achieved sub-meter accuracy (0.59–0.51 m) in moderately sized office and lab environments (141–629 m^2^), demonstrating that enriching the input data is a powerful strategy. Similarly, many of the deepest models are pipelines. For instance, the CDAE-CNN [27] and the semi-supervised DAE-CNN [33] use autoencoders to clean the noisy raw RSSI data before the main positioning task, with the latter achieving a median error of just 0.66 m. This demonstrates that the quality of the input data is as important as the positioning model itself.

The choice of architecture is also guided by the intended modeling approach. CNNs are predominantly used to model RSSI fingerprints as spatial data, treating them as 1D signals or 2D images. This approach can be highly effective, as seen in a LeNet-5-inspired model [72] that achieved 1.0–1.8 m accuracy in an 800 m^2^ area. In contrast, models like CNN-LSTM [96] or those combining CNNs with GRUs [90] explicitly model both spatial and temporal dependencies. The latter, for example, achieved a 1.04 m error in a large, multi-floor building of over 9000 m^2^, showcasing the power of temporal modeling for large-scale scenarios. DNNs, typically fully connected networks, tend to be shallower (3–8 layers) and are often applied to regression tasks. Their performance varies widely based on the environment, from 2.7 m in a 78 m^2^ lab to over 9 m in the UJIIndoorLoc dataset [24], suggesting they are more sensitive to scale and environmental complexity than specialized architectures.

In summary, the architectural analysis indicates that there is no straightforward correlation between network depth and positioning accuracy. The most effective approaches are those tailored to their specific application context. Factors such as the scale and dynamism of the environment, the availability of complementary data (e.g., RTT), and the explicit handling of signal noise through preprocessing are considerably more predictive of performance than network complexity alone. The current trend is shifting from simple, monolithic architectures toward more sophisticated, context-aware pipelines that directly address the inherent challenges of RSSI-based data.

#### 4.2.4. Architectural Analysis of CNN and DNN Models Used in BLE-Based Systems

**Figure 7:**
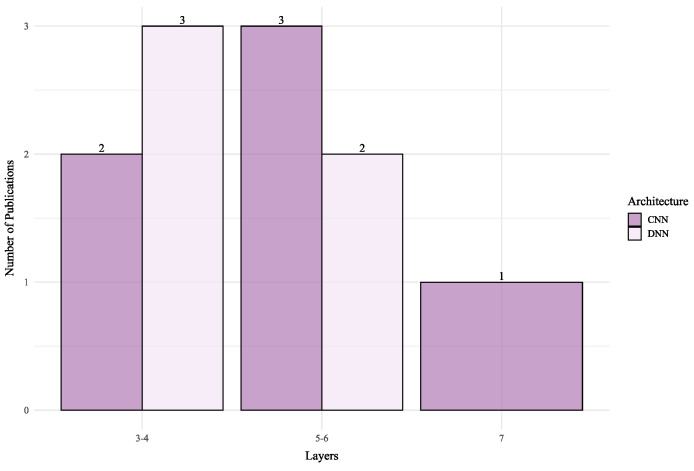
Distribution of CNN and DNN depth in BLE-based indoor positioning studies.

The application of DL in BLE RSSI systems, summarized in Table 6, follows a distinct trend driven by the technology’s constraints: a focus on lightweight, efficient architectures for small to medium-sized environments. This is reflected in the network depth (Figure 7), where BLE architectures are consistently shallower than their Wi-Fi counterparts to prioritize low latency and computational efficiency.

Shallow CNNs are the most common approach, consistently delivering strong performance. For example, a simple 1D-CNN [209] achieved an exceptional 0.25 m error in a 24 m^2^ room, while a 3D-CNN [208] reported a 0.72 m mean error in a 30 m^2^ space. Even in slightly larger areas, a baseline CNN [188,194] maintained a respectable 1.2–1.3 m error in a 72 m^2^ setup. This demonstrates that, for BLE, simple and well-tuned CNNs are highly effective in typical room or office sized deployments.

The use of DNNs and attention-based models in BLE is often geared towards specific tasks or data types. A DNN focused on movement estimation [220] achieved 0.41 m accuracy in a 40 m^2^ area. The DRVAT model [221], using a Transformer-based attention mechanism, reported a 0.88 m error in a 400 m^2^ environment, showcasing the potential of temporal modeling for improving accuracy in larger BLE deployments. These findings suggest that, while CNNs are the preferred choice for static spatial positioning, more advanced architectures are being explored to handle mobility and larger scales.

In summary, the architectural design in BLE is driven more by the application context and hardware constraints than by a push for maximum complexity. The literature shows a clear preference for shallow CNNs that provide excellent accuracy in controlled spaces. While more complex models are being explored for mobility, the overarching theme is a pragmatic balance between performance and efficiency, making CNNs the dominant choice for robust spatial feature extraction and DNNs a fast, efficient solution when computational simplicity is the priority.

### 4.3. Machine Learning Approaches Utilized in Wi-Fi CSI for Indoor Positioning (RQ2)

The analysis of Wi-Fi CSI-based systems revealed a diverse set of ML approaches, summarized in Table 7. This table summarizes the different ML algorithms identified in the selected studies, applied to indoor positioning systems based exclusively on Wi-Fi CSI. A total of 15 different types of methods were recorded, classified according to the stage of the process in which they are applied (positioning, pre-processing, aggregation, etc.), the number of appearances in the studies, and their bibliographic references. The “occurrences” column represents the number of studies in which each ML method was applied, providing a measure of the prevalence of different approaches within the Wi-Fi CSI-based indoor positioning literature.

The most frequently applied family of methods were Convolutional Neural Networks (CNNs), appearing in 23 studies [105,232,233,234,235,236,237,238,239,240,241,242,243,244,245,246,247,248,249,250,251,252,253], mainly applied for extracting spatial and temporal features from CSI matrices. Other classical algorithms such as KNN [83,230,254,255,256,257,258,259], SVM [268,273], and clustering techniques [255,284] were also identified, and these were typically used as baselines.

Several DL models beyond CNN were found, including Deep Neural Networks (DNNs) [260,261,262,263,264,265], Long Short-Term Memorys (LSTMs) [237,239,248,271], Multilayer Perceptron(MLP) [240], and even RNNs [271]. Transfer learning approaches [230,231,236,241,241] and domain adaptation or adversarial networks (GANs) [233,252,270,272] were also reported, showing a growing interest in robust methods that generalize across different environments.

Other techniques, such as ensemble methods [266,267,268,269,270], Transformer-based models [275,276], Bayesian methods [256,274], Gaussian Mixture Model (GMM) [258], and Genetic Algorithms (GA) for optimization [277], complement the landscape and are often applied to specialized scenarios or hybrid pipelines.

In summary, Wi-Fi CSI studies show a dominant trend towards DL, especially CNN-based solutions, complemented by transfer learning and domain adaptation strategies aimed at improving robustness and reducing the need for environment-specific calibration.

### 4.4. Machine Learning Approaches Utilized in Wi-Fi RTT and BLE AoA for Indoor Positioning (RQ2)

This section presents the results of Machine Learning (ML) approaches applied to Wi-Fi Round Trip Time (RTT) and Bluetooth Low Energy (BLE) Angle of Arrival (AoA) for Indoor Positioning System (IPS). Wi-Fi RTT uses time-of-flight measurements to enhance localization precision, while BLE AoA employs angular data to determine device orientation, both of which offer distinct advantages over traditional RSSI-based methods.

The analysis explores the ML techniques employed in these technologies, derived from the studies summarized in Table 8 for Wi-Fi RTT and Table 9 for BLE AoA. These results address RQ2 by highlighting the diversity and application of ML methods tailored to the unique characteristics of RTT and AoA data.

#### 4.4.1. Wi-Fi RTT-Based Indoor Positioning Systems

The analysis of the selected studies reveals a moderately diverse set of Machine Learning (ML) methods applied to Wi-Fi Round Trip Time (RTT)-based Indoor Positioning System (IPS). Deep Neural Networks (DNNs) are the most frequently used technique, appearing in six studies [285,286,287,288,289,290], due to their strong capacity to model complex spatio-temporal patterns from RTT measurements. Convolutional Neural Networks (CNNs) follow, with three occurrences [286,291,292], demonstrating their effectiveness in extracting spatial features from time-of-flight data.

Other neural-based models include Autoencoders (such as DAE, CAE, and SAE), which were employed in two studies [285,286] for feature extraction and denoising. Additionally, Recurrent Neural Networks (RNNs) [292] and Multilayer Perceptrons (MLPs) [286] were adopted, each appearing once.

Among traditional ML techniques, Random Forest (RF) and Support Vector Regression(SVR) were both used in two studies, [286,289] and [286,293], respectively. These methods provide interpretable models and strong regression capabilities. Gaussian Process Regression (GPR) also appeared in two studies [294,295], offering a probabilistic approach to positioning. Less frequently used methods include K-Nearest Neighbour (KNN) [286], Supported Vector Machine (SVM) [297], and Bayesian Networks or probabilistic methods [298], each cited once. Finally, two studies [299,300] explored alternative methods that cannot be classifiable in standard ML categories.

These results are detailed and visually summarized in Table 8.

#### 4.4.2. BLE AoA-Based Indoor Positioning Systems (IPS)

In contrast, the application of ML to Bluetooth Low Energy (BLE) Angle of Arrival (AoA)-based positioning systems remains limited, with only a few studies exploring this domain. Among these, CNNs emerge as the leading approach, appearing in two studies [210,301], primarily due to their capacity to effectively process spatial and angular information.

Other neural network architectures have been explored in isolated cases. For instance, one study applied a general neural network model [302], while another employed AnFIPNet [303], an architecture that integrates CNNs with fully connected layers and attentional filtering to enhance positioning accuracy. Additionally, a hybrid approach combining RFs and IKNNs was adopted in one study [207], demonstrating the potential of integrating ensemble and distance-based learning methods.

These methods and their corresponding studies are summarized and illustrated in Table 9.

### 4.5. Main Accuracy Improvements Achieved by Transitioning from RSSI to RTT and AoA Techniques in Wi-Fi and BLE-Based Indoor Positioning Systems (RQ3)

This section addresses RQ3 by examining the improvements in positioning accuracy achieved when transitioning from traditional RSSI-based techniques to more advanced approaches—specifically, CSI and RTT in Wi-Fi, and AoA in BLE. The analysis is organized by technology, beginning with Wi-Fi-based systems and followed by BLE, taking into account both the reported error ranges and the dimensions of the experimental environments.

#### 4.5.1. Accuracy of Wi-Fi-Based Systems Using RSSI, CSI and RTT

In the case of Wi-Fi-based indoor positioning systems, a substantial improvement is also observed when switching from traditional RSSI-based techniques to more advanced accsi and RTT-based approaches.

A total of 161 studies using RSSI, 57 using CSI and 22 using RTT were initially identified. However, not all of them were included in the quantitative comparison presented in Table 10. Specifically, 29 RSSI-based studies, 3 using CSI, and 1 RTT-based study were excluded from the statistical analysis because they did not report the mean positioning error in meters or did not provide information on the experimental area. However, these studies were retained in the broader literature review due to their scientific relevance, whether for their methodological innovation, the use of alternative evaluation metrics (such as classification accuracy), or contributions to system design and deployment.

As shown in Table 10, the systems based on Wi-Fi RSSI present errors ranging from 0.058 m to 12.4 m, in experimental areas ranging from 12.25 m^2^ to 160,000 m^2^. This large variability is influenced by the characteristics of the environment and the fluctuating nature of the signal strength.

In contrast, CSI-based systems show significantly improved and more stable results, with errors ranging from 0.15 m to 2.943 m and experimental areas from 12.4 m^2^ to 1400 m^2^. CSI leverages detailed channel state information at the subcarrier level, which captures multipath effects and phase information, enabling finer-grained positioning than RSSI.

Finally, RTT-based systems report lower and more stable errors, ranging from 0.10 m to 4.16 m, and experimental areas ranging from 24.75 m^2^ to 25,200 m^2^. RTT is based on Time of Arrival (ToA) measurement rather than signal strength, following more accurate distance estimates, especially in complex environments.

**Figure 8:**
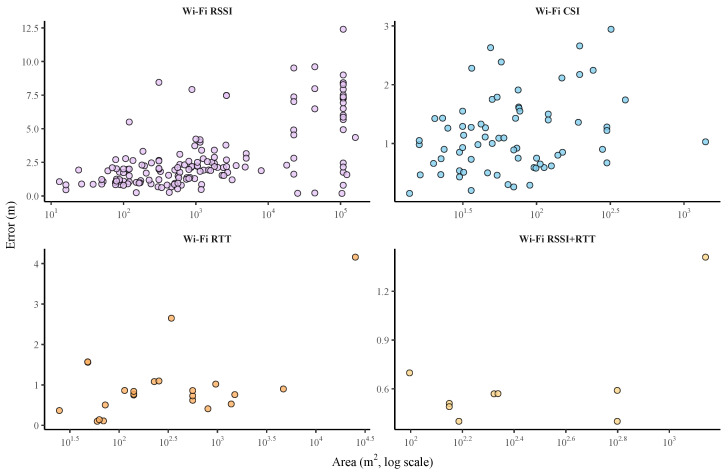
Relationship between Wi-Fi systems error and experimental area, separated by technique. The different colors indicate the Wi-Fi-based techniques employed: RSSI, CSI, RTT and RSSI+RTT.

Figure 8 illustrates the relationship between positioning error and experimental area in Wi-Fi-based systems, separated by technique. The subplot corresponding to Wi-Fi RSSI immediately stands out due to its wide vertical dispersion, with errors spanning the entire range up to 12.4 m, graphically confirming its high variance and sensitivity to environmental factors. In contrast, the subplots for Wi-Fi CSI and Wi-Fi RTT show points clustered in the low-error region, with Y-axes rarely exceeding 3–4 m. This powerful visual comparison underscores the significant and consistent improvements in accuracy offered by these advanced metrics. Furthermore, the X-axis reveals that while RSSI is applied across all scales, most CSI and RTT studies are concentrated in smaller areas, highlighting the relationship between accuracy and scale.

#### 4.5.2. Accuracy of BLE-Based Systems Using RSSI and AoA

In BLE-based indoor positioning systems, the transition from RSSI to AoA-based techniques has shown significant improvements in accuracy.

A total of 48 studies using RSSI and 5 studies using AoA were identified. Of these, only 41 RSSI-based studies were included in the comparative analysis, as they explicitly reported positioning error in meters. The remaining seven studies were excluded from this comparison because they employed alternative performance metrics—such as classification accuracy, cumulative distribution functions, or floor-level detection—that do not permit direct comparison in terms of mean positioning error. Nonetheless, these studies were retained in the overall review due to their methodological contributions or the innovative use of BLE technology.

As shown in Table 11, reported positioning errors for RSSI-based BLE systems range from 0.00023 m to 6.01 m, with experimental areas ranging from 8.24 m^2^ to 800 m^2^. This wide variability reflects the inherent limitations of RSSI, which is highly sensitive to factors such as signal attenuation, interference, and multipath propagation. Notably, the lowest error in our review, an exceptional 0.00023 m, was achieved with a Random Forest (RF) model in a controlled 35 m^2^ corridor environment [203], highlighting the high performance possible under ideal conditions.

In contrast, studies based on BLE AoA have significantly smaller errors, ranging between 0.0032 m and 1.67 m, evaluated in smaller and more controlled settings, with experimental areas ranging from 10 m^2^ to 63 m^2^. The AoA technique allows for more consistent estimates due to its ability to calculate the direction of arrival of the signal, improving the geometric robustness of the system.

**Figure 9:**
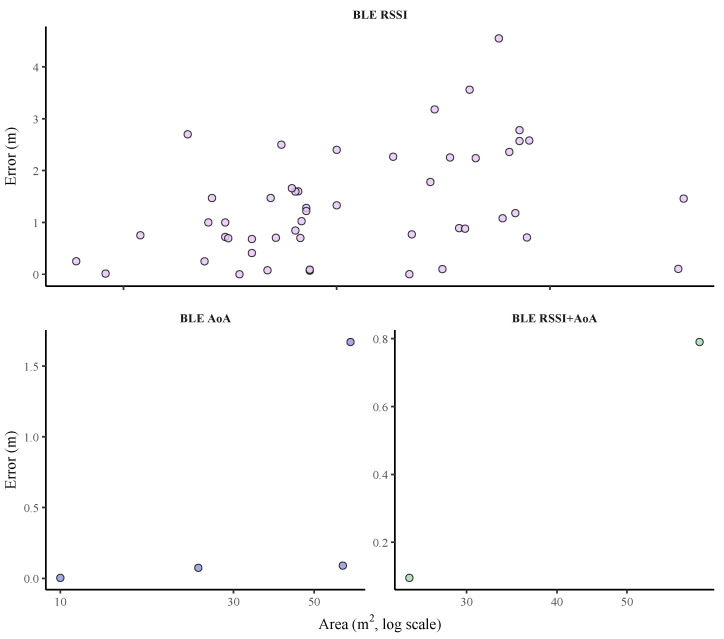
Relationship between BLE systems error and experimental area, separated by technique. The different colors indicate the BLE-based techniques employed: RSSI, AoA, and RSSI+AoA.

Figure 9 illustrates the relationship between positioning error and experimental area in BLE-based systems, separated by technique. The top panel shows that BLE RSSI results are highly dispersed, with errors reaching over 4 m. The bottom panels for BLE AoA and BLE RSSI+AoA, with their much smaller y-axis scales, dramatically illustrate the leap in precision. The results for these advanced techniques form tight, low-error clusters, with nearly all reported errors falling below 1 m. This visual contrast powerfully demonstrates the stability and accuracy gains of moving to a geometry-based principle. At the same time, the x-axis highlights the current place application of AoA, with all validations occurring in small, controlled environments under 100 m^2^.

### 4.6. Primary Challenges and Limitations Identified in Wi-Fi and BLE-Based Indoor Positioning Systems (RQ4)

Indoor Positioning System (IPS) based on Wi-Fi and Bluetooth Low Energy (BLE) have been widely studied due to their ability to leverage existing infrastructures and their compatibility with mobile devices. However, they face multiple challenges related to accuracy, stability, scalability, power consumption, security, privacy, and feasibility of implementation in real environments. This section analyzes these limitations based on a systematic review of recent literature, highlighting the main technical and practical obstacles.

One of the main challenges faced by Wi-Fi and BLE-based IPSs is their limited positioning accuracy, particularly when relying on Received Signal Strength Indicator (RSSI). Signal propagation in indoor environments is influenced by phenomena such as multipath effects, attenuation, and interference—especially in Non Line-of-Sight (NLOS) scenarios involving obstacles, furniture, and moving users. RSSI-based Wi-Fi systems report positioning errors ranging from 0.08 to 8 m, depending on the size of the environment (from 8.24 to 4240 m^2^), while BLE demonstrates slightly better accuracy, with errors between 0.00023 and 0.01 m under controlled conditions. However, the high sensitivity of RSSI to environmental variations necessitates calibration and filtering procedures, which increase system complexity and reduce scalability.

Advanced techniques such as Channel State Information (CSI) and Round Trip Time (RTT) for Wi-Fi and Angle of Arrival (AoA) for BLE have shown significant improvements, with submeter errors in controlled environments. Nevertheless, these approaches also present their own limitations. Channel State Information (CSI) provides fine-grained information about the wireless channel, enabling more accurate multipath modeling and improved positioning; however, its use is limited by the need to access raw physical-layer data, which is often restricted in commercial devices, as well as by the higher computational complexity of processing multidimensional Channel State Information (CSI) data. Round Trip Time (RTT), on the other hand, relies on IEEE 802.11mc support, restricting its adoption to modern devices and compatible access points, while Angle of Arrival (AoA) requires antenna arrays and careful calibration. These dependencies hinder the integration of Channel State Information (CSI), Round Trip Time (RTT), and Angle of Arrival (AoA)-based systems into heterogeneous multi-vendor environments and complicate their deployment in large infrastructures such as airports or shopping malls.

Other limitations include scalability and cost of implementation. Bluetooth Low Energy (BLE), although more economical and energy-efficient, has limited coverage and requires a high density of beacons to ensure accurate positioning, increasing installation and maintenance costs. Fingerprinting-based approaches also require periodic database updates, which becomes operationally intensive in dynamic environments. Power consumption remains another challenge, especially for IEEE 802.11 Wireless LAN (Wi-Fi)-based systems, which require continuous signal scanning, impacting battery life in mobile devices.

Security and privacy issues add further complexity. The continuous transmission of location information in Wi-Fi and BLE systems can expose users to risks of unauthorized tracking, especially in environments regulated by regulations such as General Data Protection Regulation (GDPR) [304], which require explicit consent and data anonymization. Wi-Fi systems are vulnerable to spoofing attacks, while BLE beacons often broadcast identifiers without encryption, and CSI-based measurements can reveal device-specific characteristics, posing new privacy risks. In addition, fingerprinting techniques store detailed information about users and physical infrastructure, requiring anonymization and encryption techniques that increase latency and system complexity.

The implementation of IPS in real environments faces significant barriers. Most of the studies analyzed have been validated in controlled environments with ideal conditions, so their performance in heterogeneous and dynamic spaces, such as hospitals or shopping centers, is not always replicable. The need for environment-specific calibrations, the lack of interoperability between standards, and user resistance due to privacy concerns or unintuitive interfaces are additional obstacles. Overcoming these barriers requires advances in ML algorithms, efficient hardware, and robust security protocols.

Despite the limitations described above, recent advances show remarkable progress toward more accurate, robust, and efficient IPS systems. Techniques that combine RSSI, CSI, RTT, and AoA, along with DL-based algorithms, are gaining interest due to their ability to mitigate reliance on manual calibrations and improve model generalization in complex environments. These solutions promise to address challenges of scalability, power consumption, and privacy, but require further research to ensure their feasibility in practical applications.

In conclusion, Wi-Fi and BLE-based IPS systems offer great potential for indoor applications, but face significant challenges in terms of accuracy, stability, scalability, power consumption, security, and practical feasibility. High signal variability, specialized hardware requirements, impact on device autonomy, and privacy concerns limit their widespread adoption. However, advances in hybrid techniques and DL algorithms represent an active line of research that could overcome these limitations, making IPS more robust and accessible for a wide range of applications in the future.

### 4.7. Least Explored Areas in the Application of Machine Learning Algorithms for Wi-Fi and BLE-Based Indoor Positioning Technologies (RQ5)

The systematic review shows that, although the use of Machine Learning (ML) in indoor positioning systems has advanced significantly, there are still key areas with a low degree of exploration. First, there is little integration of ML with Wi-Fi systems based on Channel State Information (CSI). CSI has proven to provide more detailed multipath information and higher positioning accuracy than RSSI; however, its combination with ML algorithms remains underexplored, especially in large-scale and dynamic real-world scenarios. Most CSI-based ML studies focus on controlled laboratory environments, often using modified hardware or special firmware to access physical-layer data, which limits their reproducibility in commercial deployments. Furthermore, the high dimensionality of CSI data poses additional challenges for ML models, requiring advanced preprocessing and feature extraction techniques. This gap represents a clear opportunity for research into scalable and robust ML methods for CSI-based positioning.

Secondly, there is limited exploration of of ML in Wi-Fi systems based on Round Trip Time (RTT). Although the IEEE 802.11mc standard offers significantly higher accuracy capability over RSSI-based approaches, the available scientific literature combining RTT with ML algorithms is very limited. Existing studies have mainly been developed in small-scale controlled laboratory environments, without validation in complex real-world scenarios or analysis of robustness to interference or high user density. This lack of practical adoption and generalization studies of ML models with temporal metrics such as RTT represents a clear opportunity for future research.

Thirdly, the use of ML in Bluetooth Low Energy (BLE) based systems using the Angle of Arrival (AoA) parameter remains limited. Although AoA has the potential to provide submeter accuracy by exploiting the angular information of the signal, most of the identified works focus on simulations or highly controlled laboratory environments, with no real applications or large-scale deployments. Furthermore, there is no evidence of research that combines AoA with advanced DL architectures capable of improving robustness in dynamic or high interference environments, which makes this line of research an area of great interest for the future.

Finally, the application of security and privacy oriented ML in these systems is also an area that has been little addressed. Most studies prioritize improving accuracy, leaving the protection of location data in the background. There are very few proposals that integrate privacy-preserving techniques such as federated learning or differential privacy, despite the fact that indoor location involves sensitive information susceptible to misuse.

These areas, Wi-Fi CSI and RTT with ML, BLE AoA with ML, and security and privacy in ML environments, are clear research gaps that, if addressed, could strengthen the accuracy, robustness and reliability of indoor positioning systems.

## 5. Discussion

This section synthesizes and interprets the findings presented in Section 4. The following discussion aims to provide a deeper analysis of the underlying trends and challenges in the field. By addressing the research questions defined in Section 3.2, we highlight the key insights derived from our systematic review, contextualize the evolution of ML-based positioning, and identify critical knowledge gaps.

### 5.1. Discussion on Machine Learning Methods Employed in Wi-Fi and BLE-Based Indoor Positioning Systems Using RSSI (RQ1)

This section discusses the findings presented in Section 4.2 and analyzes the ML methods used in Wi-Fi and BLE RSSI-based Indoor Positioning Systems (IPSs). The comparative study focuses on algorithm prevalence, methodological diversity, and the influence of technological constraints on model selection.

#### 5.1.1. Wi-Fi RSSI-Based Indoor Positioning Systems (IPS)

**Figure 10:**
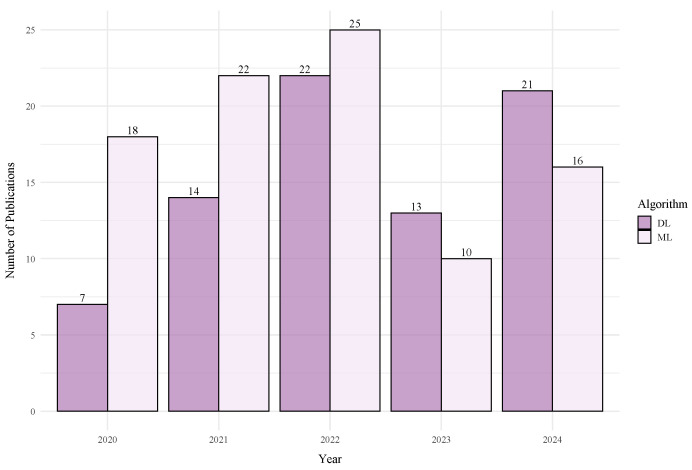
Algorithmic trends for the positioning stage in Wi-Fi RSSI-based systems (2020–2024), showing the number of unique studies per year that utilize Classical ML and Deep Learning methods.

The evolution from traditional methods to more sophisticated DL architectures is one of the most significant trends identified in this review. As illustrated in Figure 10, while classical ML algorithms remain a consistent baseline, there has been a marked increase in the adoption of DL models. The data clearly show that, since 2022, the number of studies investigating DL has nearly equaled or surpassed those focusing on classical ML, underscoring a clear paradigm shift in the field. It is important to note that this figure includes all ML/DL methods applied in the positioning process stage.

The continued prevalence of K-Nearest Neighbour (KNN) is not merely a preference but an indicator of the maturity of fingerprinting, establishing it as a robust and ubiquitous baseline against which new, more complex models are benchmarked. Its simplicity and low computational cost make it an effective starting point. However, its strong dependence on data density and vulnerability to environmental noise are precisely the limitations that have spurred the adoption of more advanced models.

The emergence of DL techniques reflects a fundamental shift towards models that can automatically learn feature representations directly for the positioning task. CNNs, in particular, are effectively adapted to treat RSSI fingerprints as 2D spatial “images,” allowing them to learn location-dependent features. LSTMs, on the other hand, model temporal sequences, making them suitable for tracking user movement. The recent inclusion of Transformer-based architectures and domain adaptation techniques (GANs) signals the latest frontier: addressing the challenge of generalization across different environments and devices.

In general, the wide range of algorithms applied reveals that there is no universally superior method. The choice depends largely on the specific characteristics of the deployment environment, the availability of labeled data, the complexity of the space, and computational constraints. The trends observed also indicate a continuous evolution of the field, from traditional methods to more sophisticated approaches that attempt to overcome the inherent limitations of RSSI as a positioning signal.

#### 5.1.2. BLE RSSI-Based Indoor Positioning Systems (IPS)

**Figure 11:**
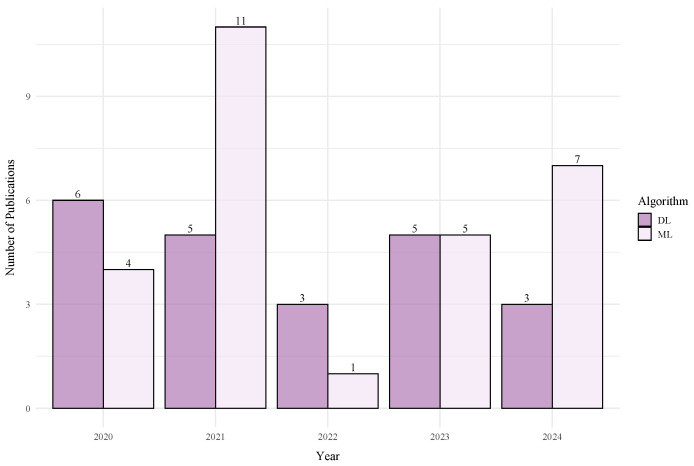
Algorithmic trends for the positioning stage in BLE RSSI-based systems (2020–2024).

In contrast to the clear paradigm shift observed in Wi-Fi, the algorithmic landscape for the BLE RSSI positioning stage remains more conservative, as visualized in Figure 11. The number of studies incorporating DL is not only lower but has also remained stable and minoritarian, without the significant growth trend seen in Wi-Fi. This visual evidence supports the conclusion that BLE research continues to prioritize lightweight, energy-efficient classical algorithms for the core positioning task.

The analysis confirms that K-Nearest Neighbour (KNN) and its variants are the undisputed backbone of BLE positioning. This is a direct consequence of the primary constraints of technology: the need for computational efficiency and low power consumption in battery-powered devices. These characteristics make lightweight, interpretable algorithms the natural choice.

While DL techniques are present, their integration for the positioning task remains limited and appears more exploratory. The scarcity of sequence modeling methods like LSTM further indicates that capturing temporal dependencies in the highly volatile BLE signal remains a significant, largely unaddressed challenge for positioning algorithms.

#### 5.1.3. Comparative Analysis Between Wi-Fi and BLE RSSI Approaches

The algorithmic divergence between Wi-Fi RSSI and BLE systems for positioning tasks, clearly illustrated by comparing the trends in Figure 10 and Figure 11, is not arbitrary. The higher data rates and more stable signal environment of Wi-Fi provide richer datasets that favor complex DL models requiring large amounts of data. In contrast, the design philosophy of BLE prioritizes low power consumption and efficiency, which naturally favors lightweight algorithms with low computational complexity.

While KNN serves as a common benchmark for both, the experimental frontier is vastly different. In Wi-Fi, the research has pushed towards advanced generalization techniques. In BLE, the innovation remains focused on optimizing the classical fingerprinting pipeline. To synthesize this comparative discussion, Table 12 provides a compact summary of the main challenges, dominant positioning models, and key research trends for both Wi-Fi and BLE RSSI-based systems.

### 5.2. Discussion on Machine Learning Approaches Utilized in Wi-Fi CSI, RTT and BLE AoA for Indoor Positioning Systems (IPS) (RQ2)

This section discusses the findings presented in Section 4.3 and Section 4.4 and provides a comparative analysis of the ML approaches employed in Wi-Fi CSI, Wi-Fi RTT, and BLE AoA-based Indoor Positioning Systems (IPSs). Differences in methodological diversity and technological constraints are analyzed, showing how each technique influences algorithm choice and research directions. The analysis focuses on how the intrinsic characteristics of each type of signal (the dimensionality of CSI, the temporal nature of RTT, and the geometric information of AoA) directly influence the choice of ML models and define the maturity of each research area.

#### 5.2.1. Wi-Fi CSI-Based Indoor Positioning Systems (IPS)

The algorithmic landscape of Wi-Fi CSI is unequivocally dominated by DL, a direct consequence of the nature of the signal. CSI provides a rich, multi-dimensional tensor of amplitude and phase information across multiple subcarriers, which is structurally analogous to an image. This explains the clear predominance of Convolutional Neural Networks (CNNs), as they are expertly designed to process such spatial or time-frequency representations and automatically extract potent, location-specific features that are nearly impossible to model with traditional algorithms. Deep Neural Networks (DNNs) and Long Short-Term Memorys (LSTMs) further extend this by capturing complex, non-linear relationships and temporal dependencies, respectively.

This focus on advanced models marks a clear departure from RSSI-based research. While RSSI systems still rely heavily on KNN, the CSI field has largely moved on to sophisticated architectures like Transformers and domain adaptation techniques (GANs). This shift is driven by necessity: the high sensitivity of CSI to specific hardware and environments makes generalization a critical problem. As a result, classical algorithms like KNN and SVM appear in the CSI literature almost exclusively as performance benchmarks, rather than as primary solution candidates.

However, the high dimensionality of CSI, which enables high accuracy, also poses challenges in terms of computational overhead and dimensionality. This explains why our review found consistent use of autoencoders for dimensionality reduction and noise removal, highlighting that robust preprocessing is a fundamental prerequisite for successful CSI-based positioning. This technical barrier, combined with the limited availability of commercial devices that expose raw CSI data, currently limits its large-scale adoption, despite its proven sub-meter accuracy in controlled environments.

#### 5.2.2. Wi-Fi RTT-Based Indoor Positioning Systems (IPS)

Unlike the image-like data of CSI, Wi-Fi RTT provides numerical ToA measurements, leading to a more balanced and diverse algorithmic landscape. The predominance of DNNs and CNNs reflects their powerful ability to model the complex, non-linear relationship between RTT measurements and true distance, especially in multipath-rich environments.

However, traditional ML methods like Random Forest (RF) and SVR retain significant relevance. This is because the main task is fundamentally a regression problem with more interpretable features (time/distance) than raw CSI. These classical models offer excellent performance with faster training and greater interpretability, making them a pragmatic choice for many scenarios.

A key finding in the RTT literature is the common use of probabilistic models like GPR. This shows that researchers are interested in more than just predicting a location; they also want to quantify the uncertainty of that prediction. Knowing how reliable a location estimate is can be critical for navigation, especially since real-world ToA measurements can be noisy. The use of these uncertainty-aware models, alongside a variety of other established methods, suggests that RTT-based positioning is a relatively mature and versatile research area.

#### 5.2.3. BLE AoA-Based Indoor Positioning Systems

Research on BLE AoA represents the most emerging stage among advanced metrics, with an algorithmic landscape that is still emerging. The limited number of studies naturally restricts methodological diversity, but a clear pattern is already emerging: CNNs are the predominant choice. This is a logical starting point, as the angular information derived from AoA antenna arrays can be processed as image-like data, making convolutional layers an effective tool for feature extraction.

Despite the focus on CNNs, early signs of exploration are already visible. The introduction of attention-based architectures (AnFIPNet) suggests a trend toward more sophisticated DL, while the use of hybrid models such as RF + IKNN indicates that combining classifiers with distance-based algorithms remains a viable strategy, especially in scenarios with limited data. Overall, the current literature shows that ML applications in BLE AoA remain exploratory, with significant scope for the development and validation of more diverse and robust models.

#### 5.2.4. Comparative Analysis Between Wi-Fi CSI, RTT and BLE AoA

The comparative analysis reveals that Wi-Fi CSI, Wi-Fi RTT, and BLE AoA each have distinct research profiles and levels of maturity, shaped by their signal characteristics, data availability, and deployment challenges.

- Wi-Fi CSI is the most methodologically sophisticated, heavily reliant on complex, data-hungry architectures (CNNs, Transformers, GANs) to harness its high-dimensional data. Its development stage is advanced, but it faces the most significant practical deployment hurdles.
- Wi-Fi RTT leverages a balanced mix of traditional regression models (RFs, SVRs) and DL (DNNs). The numerical nature of its data makes it amenable to both approaches, and the strong presence of probabilistic models like GPRs indicates a mature focus on reliability.
- BLE AoA is the most emerging of the three, its algorithmic landscape is the least diverse. The research is currently dominated by CNNs as a natural fit for processing geometric data, with the field still in an active, experimental phase.

Despite their differences, a powerful point of convergence is the universal adoption of CNNs in all three techniques, underscoring its versatility in learning spatial features from various types of data, whether CSI matrices, RTT time series, or AoA angular patterns. However, the key differences offer a clear picture of three technologies at different stages of their research lifecycle, each with its own set of challenges and opportunities.

### 5.3. Discussion on Main Accuracy Improvements Achieved by Transitioning from RSSI to RTT and AoA Techniques in Wi-Fi and BLE-Based Indoor Positioning Systems (IPS) (RQ3)

One of the clearest findings of this systematic review is the substantial and consistent accuracy improvement gained by transitioning from RSSI to advanced signal metrics. However, our analysis reveals that this performance gain comes with significant trade-offs in terms of deployment complexity, hardware dependency, and the generalizability of the reported results. This section dissects this critical accuracy-practicality balance.

#### 5.3.1. Discussion on the Accuracy of Wi-Fi-Based Systems Using RSSI, CSI and RTT

The transition from RSSI to CSI and RTT in Wi-Fi systems yields undeniable improvements in positioning accuracy. The reason is fundamental: these techniques leverage more robust physical properties of the signal.

- CSI achieves lower and more stable errors by exploiting fine-grained channel information, allowing for better modeling of multipath effects and reducing sensitivity to environmental fluctuations.
- RTT, by measuring the signal’s Time of Flight (ToF) rather than its intensity, provides inherent robustness against signal power variations and interference, leading to stable sub-meter or few-meter accuracy in dynamic scenarios.

However, this superior accuracy must be contextualized. Our review shows that CSI and RTT-based experiments are predominantly conducted in smaller, more controlled environments (labs, offices), whereas RSSI-based studies cover a much wider and more challenging range of large-scale deployments (malls, universities). While the accuracy improvement is primarily due to the superior measurement principles, the impressive sub-meter figures reported for CSI and RTT are also a reflection of these controlled testbeds.

Furthermore, these techniques face significant deployment barriers. CSI requires access to raw physical-layer data, which is not exposed by most commercial Wi-Fi chipsets. RTT, in turn, depends on IEEE 802.11mc compatibility, limiting it to modern devices and access points. These hardware dependencies explain why RSSI, despite its flaws, remains the most widely implemented method due to its simplicity and backward compatibility.

#### 5.3.2. Discussion on the Accuracy of BLE-Based Systems Using RSSI and AoA

A similar trend is observed in BLE, where AoA offers a leap in accuracy over RSSI. By leveraging the direction of arrival of the signal, AoA-based systems provide geometrically robust estimates, and most of the studies reviewed achieve sub-meter or even decimeter accuracy. This makes AoA a very promising technique for high-precision applications.

However, this impressive accuracy is currently largely limited to a very specific context. The vast majority of studies on AoA are conducted in small, controlled spaces (often <100 m^2^) with specialized antenna arrays. This controlled environment minimizes signal scattering and simplifies position calculation, contributing to the excellent results. Therefore, although the potential of AoA is evident, the generalization of its performance to large, complex, and dynamic real-world scenarios remains an open and critical research question. The technical and economic challenges posed by the deployment and calibration of the necessary specialized hardware are significant obstacles to its widespread adoption.

#### 5.3.3. Comparative Analysis Between the Accuracy of Wi-Fi and BLE Technologies

The results of this review do not suggest that one technology is universally better than another. On the contrary, they highlight a clear range of options, each with an optimum point defined by the balance between accuracy requirements and practical constraints. Therefore, the transition from RSSI to more sophisticated techniques is a matter of choosing the right tool for a specific application context.

- RSSI remains the pragmatic choice for low-cost, large-scale applications where sufficient accuracy at the room or zone level is sufficient and leveraging existing infrastructure is a priority.
- BLE AoA is the high-accuracy choice for specialized, small-scale use cases where decimeter-level accuracy is critical and the cost of dedicated hardware and a controlled environment is justifiable.
- Wi-Fi CSI excels in complex indoor environments where multipath is a major challenge. It is best suited for applications where high accuracy is required and where the hardware can be controlled to extract the necessary channel data.
- Wi-Fi RTT is emerging as the most promising scalable, high-precision solution for the future. It offers a great balance between robustness and ease of integration, as more and more commercial devices and access points adopt the IEEE 802.11mc standard.

In conclusion, although going beyond RSSI offers substantial improvements in positioning accuracy, it requires careful evaluation of the specific needs of the application against the costs and limitations of each advanced technology.

### 5.4. Discussion on Performance of ML Models in Different Environmental Topologies

A crucial aspect of this review, synthesized from the data presented in Table 10 and Table 11, is to assess how the performance of ML models varies across different real-world environments. While a direct quantitative comparison is impeded by study heterogeneity, our analysis reveals distinct trends regarding the suitability of different ML architectures for specific topologies:

- Structured Office and Laboratory Environments: These are the most common testbeds, characterized by stable signal propagation and typically smaller areas. In these controlled conditions, models can achieve their highest accuracy. We found numerous examples of sub-meter performance, such as a 1D-CNN achieving a remarkable 0.058 m error in a 37.6 m^2^ space [92]. The absolute best-case result in our review was found here: a Random Forest (RF) model for BLE RSSI reported an exceptional 0.00023 m error in a 35 m^2^ corridor [203]. These results demonstrate that under ideal conditions, ML can achieve near-perfect accuracy, suggesting that the main challenge is not the models themselves, but their robustness to environmental changes.
- Dynamic, Large-Scale Public Spaces (Malls, University Campus): As the scale and dynamism of the environment increase, performance degrades significantly. This is most evident in studies validated on large-scale public benchmark datasets like UJIIndoorLoc (108,703 m^2^), Tampere University (22,570 m^2^), or custom deployments in areas up to 160,000 m^2^ [100]. In these scenarios, reported errors frequently exceed 5 m, with some reaching as high as 12.4 m [27]. The literature indicates that models capable of capturing temporal dependencies, like LSTMs and Transformers, offer superior robustness here. For example, an Attention-guided LSTM [130] achieved a 2.58 m error in a large, heterogeneous 800 m^2^ environment, outperforming purely static models.
- Harsh Industrial Environments (Warehouses, Factories): These topologies with severe multipath are the most challenging. Here, model robustness is more critical than sheer complexity. Tree-based ensemble methods like Random Forest and XGBoost often show reliable performance due to their ability to handle noisy and outlier data. While fewer studies explicitly target these environments, a hybrid RF+IKNN model for BLE AoA achieved 0.4214 m accuracy in a cluttered lab [207], demonstrating the power of combining robust classifiers with geometric data in challenging environments. Achieving consistent accuracy below 3 m is considered a strong result in these settings.

In summary, our review indicates that there is no single best ML model. The optimal choice is highly context-dependent, defined by a trade-off between the desired accuracy and the complexity of the operational environment. Simple CNNs excel in stable labs, LSTMs are essential for robustness in large, dynamic spaces, and robust ensemble methods are a pragmatic choice for the harshest industrial settings.

### 5.5. Discussion on Practical Deployment Challenges and Considerations

Beyond algorithmic performance, the practical viability of Wi-Fi and BLE-based IPSs is dictated by several real-world deployment challenges identified throughout our review. These considerations often determine the choice of technology and methodology for a given application:

- Deployment and Maintenance Costs: A primary advantage of Wi-Fi-based systems is their ability to leverage existing network infrastructure, minimizing initial hardware costs. However, this is often offset by the significant hidden cost of creating and maintaining radio maps. The upkeep of these radio maps is a major operational load, as they require frequent recalibration when the physical environment changes (furniture is moved). BLE systems, while requiring the purchase and installation of beacons, introduce the recurring cost of battery replacement and hardware maintenance.
- Calibration Effort: The majority of high-accuracy systems reviewed, particularly those based on RSSI fingerprinting, rely on a time-consuming and labor-intensive calibration phase. This process, which involves collecting detailed signal measurements at numerous known locations, represents a significant barrier to scalability. The high initial effort makes deploying such systems in large, multi-floor buildings prohibitively expensive and slow, which explains the strong research interest in calibration-free or calibration-light methods.
- Scalability and Energy Consumption: Scalability is not only limited by calibration but also by operational factors. In BLE systems, achieving high accuracy often requires a high density of beacons, increasing both hardware and maintenance costs. For the end-user device, energy consumption is a critical concern. Continuous Wi-Fi or BLE scanning for positioning purposes can significantly drain the battery of mobile devices, impacting user experience. This trade-off between positioning update frequency (and thus, real-time accuracy) and battery life remains a key challenge, particularly for applications requiring continuous tracking or roaming between different areas.

In summary, while the literature often focuses on improving accuracy, these practical challenges related to cost, labor, and energy consumption are frequently the deciding factors in real-world adoption. Future research must therefore increasingly focus on solutions that are not only accurate but also scalable, cost-effective, and sustainable to maintain.

### 5.6. Discussion on Benchmarking and Reproducibility

A key finding of this systematic review is not just the evolution of ML methods but the persistent and serious deficiency of a shared assessment framework in the indoor positioning field. Although numerous studies report impressive sub-meter accuracy, it is nearly impossible to compare these approaches directly and fairly. As our analysis shows, results are frequently obtained under highly specific, controlled, and heterogeneous conditions that are rarely replicated.

This problem, which is frequently disregarded, stands in the way of both scientific advancement and practical application. Our analysis shows that heterogeneity exists on multiple levels: the size, shape, and construction materials of the experimental area, as well as human traffic and environmental dynamics, can all significantly affect performance metrics. An algorithm designed for a 50 m^2^ static office is probably not going to work as well in a 10,000 m^2^ dynamic shopping center with multiple floors.

The use of different metrics, such as mean error, median error, 75th percentile error, or room-level accuracy, prevents a standardized comparison of positioning accuracy. While a few widely adopted, large-scale benchmark datasets exist, such as UJIIndoorLoc, Tampere University, and UTSIndoorLoc, our review shows that a significant portion of studies still rely on generating their own smaller, private datasets. This practice, while necessary for exploring novel scenarios, perpetuates the problem of non-comparability and makes it difficult to fairly assess the true progress in the field.

As a consequence, some reported results may overestimate practical performance or fail to generalize outside the experimental setting. To advance the field, there is a pressing need for standardized evaluation protocols and best practices for benchmarking, including clearly defined datasets, reproducible experimental conditions, and consistent reporting of metrics. Establishing such guidelines would not only improve comparability but also strengthen the credibility and practical relevance of research outcomes.

## 6. Conclusions and Future Directions

This paper presents a systematic review of the literature on indoor positioning systems based on ML using Wi-Fi and BLE technologies, encompassing research published between 2020 and 2024. Following the PRISMA methodology, a total of 280 studies were analyzed and classified according to the signal metrics employed (RSSI, CSI, RTT, and AoA), the ML methods were applied, and the evaluation parameters were reported.

The analysis reveals that Wi-Fi remains the dominant technology and that RSSI-based digital fingerprinting continues to be widely studied due to its simplicity and compatibility with existing infrastructures. However, its known sensitivity to environmental dynamics and multipath have driven growing research interest in advanced measurements such as CSI, RTT, and AoA. The combination of CSI with deep neural networks, RTT with probabilistic and regression-based models, and BLE AoA with angle-based estimation approaches has shown remarkable improvements in accuracy, often achieving submeter accuracy under controlled conditions.

From a methodological perspective, this review reveals a clear evolution from traditional algorithms—such as KNN, SVM, and decision trees—toward DL-based approaches, particularly CNNs, DNNs, LSTMs, and hybrid models. These techniques have demonstrated greater capacity to capture complex spatio-temporal dependencies in noisy indoor environments. However, the reported results remain highly heterogeneous across studies, as error metrics, implementation scales, and experimental conditions vary substantially, which limits the comparability and generalization of findings.

Despite these advances, several challenges remain unresolved. Current IPS research continues to face issues of scalability in large dynamic environments, high calibration costs, device heterogeneity, and the absence of standardized open datasets for fair benchmarking. These limitations prevent the smooth transition of many promising solutions from controlled testbeds to large-scale real-world deployments. Looking ahead, several promising directions for future research can be identified:

- Advances in research on high-precision metrics: It is a priority to expand studies on ML regarding BLE AoA and Wi-Fi RTT. These metrics represent a minority of the literature, despite their proven advantages in terms of precision. Future work should validate their performance in large-scale dynamic environments and focus on developing lightweight and generalizable models suitable for implementation in commercial mobile and IoT devices.
- Addressing data heterogeneity and standardization: A critical barrier is the absence of standardized, open, large-scale datasets, particularly for CSI, RTT, and AoA. It is essential to prioritize their creation to enable fair benchmarking and train robust and adaptable ML models capable of functioning in changing environments without the need for costly recalibrations.
- ML that preserves security and privacy: A crucial and under-explored area is the application of ML to protect location data. Future research should integrate techniques such as Federated Learning or Differential Privacy directly into IPS algorithms to ensure user privacy and compliance with regulations such as the GDPR, going beyond accuracy as the sole optimization metric.
- Multimetric fusion: Research into hybrid and multimetric systems that combine RSSI, CSI, RTT, and AoA is a promising frontier. The challenge lies in designing sophisticated fusion algorithms, likely based on DL, that can intelligently weight the contribution of each metric to leverage their complementary strengths and mitigate their individual weaknesses.
- Optimization: The design of lightweight architectures for real-time implementation on mobile devices and the IoT remains a key challenge. Future research should explore techniques for creating efficient models that ensure low latency and low energy consumption without significantly sacrificing accuracy.

Furthermore, the evolution of wireless standards (Wi-Fi 802.11az and BLE 5.1) and the emergence of 6G communications will provide the technical basis for a paradigm shift. Future 6G networks are expected to feature Integrated Sensors and Communications (ISAC), where positioning becomes a native network service. Technologies such as Terahertz (THz) communication and large-scale antenna arrays (ELAAs)/massive MIMO will offer unprecedented bandwidth and spatial resolution, enabling centimeter-accurate localization. The research challenge will be to develop novel methods based on ML, such as distributed or federated learning models, that can process the enormous amounts of data generated by these systems in real time while preserving user privacy and adapting to the unique characteristics of the THz band channel.

In conclusion, while RSSI-based solutions remain the backbone of Wi-Fi and BLE positioning, the transition to advanced measurements and DL methods signals a paradigm shift in this field. Continued efforts to expand research on ML in less explored signal metrics, such as AoA and RTT, along with advances in robustness, scalability, and standardization, will be essential to unlocking the full potential of ML-based IPS in real-world applications.

## Figures and Tables

**Table 1:** Comparison of existing reviews on Indoor Positioning Systems.

Work	Years Covered	BLE RSSI	Wi-Fi RSSI	Wi-Fi RTT	BLE AoA	Wi-Fi CSI	ML	DL	Applications
Ding et al. [7]	2008–2013	✗	✓	✗	✗	✗	✓	✗	✗
Sakpere et al. [8]	2000–2017	✗	✗	✗	✗	✗	✗	✗	✗
Tariq et al. [9]	N/S–2018	✓	✓	✗	✗	✗	✓	✗	✗
Zafari et al. [10]	2005–2018	✓	✓	✗	✗	✗	✓	✗	✓
Obeidat et al. [11]	2010–2021	✓	✓	✗	✗	✗	✓	✗	✓
Singh et al. [12]	2015–2021	✗	✓	✗	✗	✗	✓	✓	✓
Sesyuk et al. [13]	2014–2022	✓	✓	✗	✗	✗	✓	✓	✓
Feng et al. [14]	2016–2022	✗	✗	✗	✗	✓	✗	✓	✓
Simelane et al. [15]	2016–2023	✓	✓	✗	✗	✗	✓	✓	✓
Dai et al. [16]	2017–2023	✗	✓	✓	✗	✗	✓	✓	✗
Park et al. [17]	2018–2023	✗	✓	✗	✗	✗	✓	✗	✗
Bastiaens et al. [18]	2007–2024	✗	✗	✗	✗	✗	✗	✗	✓
Morgan [19]	2010–2024	✓	✗	✓	✗	✗	✓	✗	✓
Singh et al. [20]	2010–2024	✓	✓	✗	✗	✓	✓	✗	✓
Lin et al. [21]	2015–2024	✗	✓	✗	✗	✓	✓	✓	✓
Shi and Gong [22]	2018–2024	✓	✗	✓	✗	✗	✓	✗	✓
Ahmad et al. [23]	2018–2024	✗	✗	✗	✗	✓	✗	✓	✓
Our Review	2020–2024	✓	✓	✓	✓	✓	✓	✓	✓

✓ = Included ✗ = Not included

**Table 2:** Scopus and Web of Science search queries, where the symbol ‘*’ stands for a wildcard.

Database	Query	Documents
Web of Science	TS = (“Indoor Position*” OR “Indoor Track*” OR “Indoor Locati*” OR “Indoor Locali*” OR “Indoor Navigat*”) AND TS = (“BLE” OR “Bluetooth Low Energy” OR “Wi-Fi” OR “WLAN” OR “Wifi” OR “802.11”) AND TS = (“RTT” OR “Round-trip time” OR “round trip time” OR “AoA” OR “Angle of arrival” OR “Direction of Arrival” OR “DoA” OR “RSS” OR “RSSI” OR “Received signal strength” OR “CSI” “Channel state information”) AND TS = (“Machine Learning” OR “ML” OR “Deep Learning” OR “DL” OR “Neural Networks” OR “NN” OR “KNN” OR “K-nearest” OR “SVM” OR “Support Vector Machine”) and 2020 or 2021 or 2022 or 2023 or 2024 (Publication Years)	537
SCOPUS	( TITLE-ABS-KEY ( “Indoor Position*” OR “Indoor Track*” OR “Indoor Locati*” OR “Indoor Locali*” OR “Indoor Navigat*” ) AND TITLE-ABS-KEY ( “BLE” OR “Bluetooth Low Energy” OR “Wi-Fi” OR “WLAN” OR “Wifi” OR “802.11” ) AND TITLE-ABS-KEY ( “RTT” OR “Round-trip time” OR “round trip time” OR “AoA” OR “Angle of arrival” OR “Direction of Arrival” OR “DoA” OR “RSS” OR “RSSI” OR “Received signal strength” OR “CSI” OR “Channel state information” ) AND ALL ( “Machine Learning” OR “ML” OR “Deep Learning” OR “DL” OR “Neural Networks” OR “NN” OR “KNN” OR “K-nearest” OR “SVM” OR “Support Vector Machine” ) ) AND PUBYEAR > 2019 AND PUBYEAR < 2025	1081

**Table 3:** Machine Learning Methods in systems based on Wi-Fi RSSI.

Machine Learning Method	Process Stage	Occurrences	Study References
Autoencoders (DAE, CAE, AE, SSAE)	Preprocessing	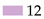	[24,25,26,27,28,29,30,31,32,33,34,35]
K-Nearest Neighbors	Positioning	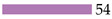	[36,37,38,39,40,41,42,43,44,45,46,47,48,49,50,51,52,53,54,55,56,57,58,59,60,61,62,63,64,65,66,67,68,69,70,71,72,73,74,75,76,77,78,79,80,81,82,83,84,85,86,87,88,89]
Convolutional Neural Network		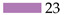	[25,27,29,31,33,72,90,91,92,93,94,95,96,97,98,99,100,101,102,103,104,105,106]
Random Forest		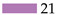	[40,41,46,48,52,55,57,61,62,82,107,108,109,110,111,112,113,114,115,116,117]
Long Short-Term Memory		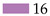	[32,35,53,118,119,120,121,122,123,124,125,126,127,128,129,130]
Deep Neural Network		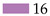	[24,26,34,116,131,132,133,134,135,136,137,138,139,140,141,142]
Support Vector Machine		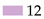	[40,52,57,59,61,62,116,127,143,144,145,146]
Multilayer Perceptron		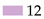	[28,59,62,123,134,140,147,148,149,150,151,152]
Ensemble Methods		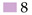	[66,142,150,151,152,153,154,155]
XGBoost		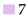	[40,74,107,108,144,156,157]
Gaussian Process Regression		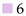	[48,50,158,159,160,161]
Bayesian Networks/Methods		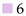	[153,154,162,163,164,165]
Genetic Algorithms and Optimization		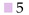	[136,150,165,166,167]
Domain Adaptation/Adversarial Networks		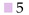	[30,168,169,170,171]
Decision Tree		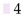	[41,52,62,111]
Support Vector Regression		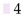	[47,48,111,172]
Transformer-based Methods		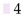	[173,174,175,176]
Recurrent Neuronal Network		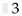	[125,177,178]
Gradient Boosting		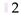	[41,156]
Gaussian Mixture Model		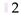	[114,179]
Capsule Networks		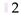	[180,181]
Naive Bayes		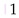	[55]
Other Methods		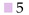	[65,105,155,182,183,184]
Clustering (K-means, DBSCAN, HDBSCAN, RPCA)	Aggregation	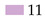	[44,45,49,64,65,89,106,119,124,158,163]

**Table 4:** Machine Learning Methods in systems based on BLE RSSI.

Machine Learning Method	Process Stage	Occurrences	Study References
Autoencoders (DAE, CAE, AE)	Preprocessing	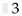	[185,186,187]
K-Nearest Neighbors	Positioning	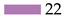	[63,186,188,189,190,191,192,193,194,195,196,197,198,199,200,201,202,203,204,205,206,207]
Convolutional Neural Network		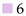	[186,188,194,208,209,210]
Other Neural Networks (BPNN, FNN...)		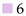	[185,196,211,212,213,214]
Multilayer Perceptron		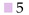	[189,203,215,216,217]
Random Forest		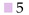	[191,199,201,203,207]
Support Vector Regression		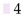	[189,196,218,219]
Deep Neural Network		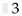	[192,220,221]
Support Vector Machine		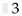	[200,203,218]
Gaussian Process Regression		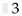	[206,219,222]
Feed-Forward Neural Network		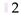	[187,208]
Naive Bayes		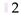	[194,199]
Trilateration-based Methods		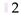	[189,223]
XGBoost		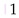	[201]
Boosted/Bagged Trees		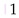	[222]
Decision Tree		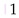	[200]
Hidden Markov Model		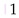	[192]
Particle Swarm Optimization		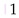	[224]
Principal Component Analysis		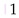	[225]
Long Short-Term Memory		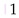	[130]
Transformer-based Methods		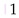	[221]
Other Methods		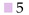	[184,219,226,227,228]
K-Means	Aggregation	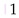	[229]

**Table 5:** Summary of CNN and DNN architectures for Wi-Fi RSSI-based Indoor Positioning Systems.

Study Reference	DL Architectures	Details of the Architecture	Depth
[72]	CNN	LeNet-5: 2 conv. + 2 pool. + 3 FC	7 layers
[90]	CNN	3 submodules: 2 conv. + multi-scale + BN + pool.	8 layers
[91]	CNN	2–3 conv. + 1–2 pool. + 15 FC + softmax; dropout, BN	18–20 layers
[25]	CNN	2 conv. + 2 FC + symmetric deconv. in decoder	4–6 layers
[92]	CNN	2 conv. + 2 pool. + 1 FC (softmax)	4 layers
[93]	CNN	3 conv. + 2 pool. + flatten + 3 FC	8 layers
[29]	CNN	1D-CNN: 4 conv. + pool. + FC	6–9 layers
[31]	CNN	1D-CNN: 4 conv. + 2–3 pool. + FC + ELM-AE	7–9 layers
[94]	CNN	1D-CNN + SAE; classification and regression branches	7 layers
[95]	CNN	2D-CNN: 3 conv. + 3 pool. + 1 FC; with EfficientNetV2	7 layers
[96]	CNN	CNN (2 conv. + 2 pool. + 1 FC) + LSTM (1 + 1 FC)	7 layers
[97]	CNN	Wavelet-CNN: 2–3 conv. + 2–3 pool. + 1 FC; BN	7–10 layers
[99]	CNN	1D-CNN: 5 conv. + 5 pool. + 5 BN + 2 FC + softmax	18 layers
[101]	CNN	2D-CNN: 2 conv. + pool. + flatten + 2 FC + 3 BN	9 layers
[102]	CNN	3 models: conv. + FC; output: softmax or linear	Not specified
[103]	CNN	Fully convolutional: 8 conv. layers (64 filters, ReLU)	8 layers
[104]	CNN	CIFAR-10-style CNN: 3 conv. + 3 pool. + 2 FC + softmax	13 layers
[27]	CNN	CDAE-CNN: CDAE (3 conv. + pool. + deconv., dropout) + CNN (4–7 conv., pool., flatten, FC)	16–21 layers
[33]	CNN	DAE-CNN (semi-supervised): DAE (3 conv. + 3 pool. + 3 deconv. + upsampling) CNN (4 conv. + 4 pool. + upsampling + FC)	21 layers
[106]	CNN	3 Conv2D + 2 MaxPooling + 3 FC (3072, 1024, 74)	8 layers
[116]	DNN	4 FC layers (128, 64, 32, 1 nodes); input: LOS/NLOS features	4 layers
[131]	DNN	DRL (DDQN): 3 FC layers (256, 128, 8); Q-values for 3D bisection	3 layers
[24]	DNN	SAE (3 FC) + DNN with 7 hidden FC + output; softmax regression	8 layers
[132]	DNN	5 FC (500 nodes, ReLU) + output FC (2 nodes, linear)	6 layers
[133]	DNN	Attention layer + two DNN branches (3 FC each + output)	5 layers
[134]	DNN	PDF-BPNN: 2 hidden FC + output	4 layers
[135]	DNN	RandNN-IAUKF: 2–3 hidden FC + output; IAUKF filtering	5 layers (estimated)
[137]	DNN	3 hidden FC layers; regression output	5 layers
[26]	DNN	DIFF with ReLU and softmax; architecture not detailed	Not specified
[138]	DNN	GAN (1 hidden FC, sigmoid) + DNN (2–3 hidden FC, ReLU)	GAN: 3 layers; DNN: 4–5 layers
[139]	DNN	1 hidden FC (220 nodes, ReLU); Xavier init.; RMSE loss	3 layers
[141]	DNN	DCCA: 2 fully connected DNNs (sigmoid); + MLP with ReLU	DCCA: 3 layers each; MLP: 3 layers
[34]	DNN	SSAE: 2 hidden FC (100–200 nodes); linear output regression	4 layers
[142]	DNN	DNN (1 input, 2 hidden, 1 output)	4 DNN layers

**Table 6:** Summary of CNN and DNN architectures for BLE RSSI-based Indoor Positioning Systems.

Study Reference	DL Method	Architecture	Depth
[188]	CNN	2 conv. + 1 FC + output	4 layers
[208]	CNN	Autoencoder: encoder + 1 latent + 1 decoder	3 layers
[209]	CNN	2 conv. + max-pooling + dropout + 1 FC + softmax	5 layers
[210]	CNN	Dual-branch CNN: each branch with 2 conv. + 2 pooling + 2 FC + softmax	7 layers per branch
[192]	DNN	4 hidden FC layers + softmax output	5 layers
[220]	DNN	4 hidden FC layers + 4 outputs	5 layers
[221]	DNN	1 embedding + 2 encoders + 1 output	4 layers
[194]	CNN and DNN	DNN: FC CNN: input, 2 conv. + 2 pooling + FC + output	DNN: 3 layers; CNN: 6 layers
[186]	CNN and DNN	DNN (RSS-NN): 1 hidden FC + output CNN: 2 conv. + 2 pooling + flatten + FC + output	DNN: 3 layers; CNN: 6 layers

**Table 7:** Machine Learning methods in systems based on Wi-Fi CSI.

Machine Learning Method	Process Stage	Occurrences	Study References
Transfer Learning	Preprocessing	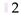	[230,231]
Convolutional Neural Network	Positioning	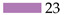	[105,232,233,234,235,236,237,238,239,240,241,242,243,244,245,246,247,248,249,250,251,252,253]
K-Nearest Neighbors		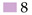	[83,230,254,255,256,257,258,259]
Deep Neural Network		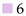	[260,261,262,263,264,265]
Ensemble Methods		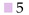	[266,267,268,269,270]
Long Short-Term Memory		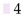	[237,239,248,271]
Domain Adaptation/Adversarial Networks		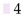	[233,252,270,272]
Transfer Learning		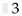	[236,241,248]
Support Vector Machine		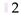	[268,273]
Bayesian Networks/Methods		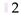	[256,274]
Transformer-based Methods		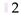	[275,276]
Multilayer Perceptron		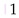	[240]
Recurrent Neuronal Network		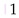	[271]
Gaussian Mixture Model		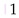	[258]
Genetic Algorithms		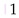	[277]
Other Methods		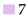	[231,278,279,280,281,282,283]
Clustering (K-means, DBSCAN, HDBSCAN)	Aggregation	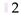	[255,284]

**Table 8:** Machine Learning methods in systems based on Wi-Fi RTT.

Machine Learning Method	Process Stage	Occurrences	Study References
Autoencoders (DAE, CAE, AE)	Preprocessing	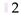	[285,286]
Deep Neural Network	Positioning	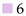	[285,286,287,288,289,290]
Convolutional Neural Network		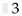	[286,291,292]
Random Forest		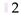	[286,289]
Support Vector Regression		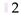	[286,293]
Gaussian Process Regression		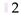	[294,295]
K-Nearest Neighbors		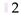	[286,296]
Multilayer Perceptron		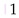	[286]
Recurrent Neural Network		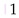	[292]
Support Vector Machine		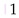	[297]
Bayesian Networks/Methods		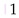	[298]
Other Methods		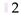	[299,300]

**Table 9:** Machine Learning Methods in systems based on BLE AoA.

Machine Learning Method	Process Stage	Occurrences	Study References
Convolutional Neural Network	Positioning	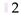	[210,301]
Neural Network		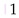	[302]
AnFIPNet		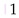	[303]
Random Forest + Improved KNN		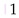	[207]

**Table 10:** Comparison of positioning accuracy in Wi-Fi-based systems using RSSI, CSI, and RTT techniques.

Technique	Studies	Error Range (m)	Experimental Area (m^2^)	Environmental Context
RSSI	132	0.058 m–12.4 m	12.25 m^2^–160,000 m^2^	Highly Diverse (Labs, Offices, Malls, Large Public Buildings)
CSI	54	0.15 m–2.943 m	12.4 m^2^–1400 m^2^	Controlled Environments (Primarily Labs and Offices)
RTT	21	0.10 m–4.16 m	24.75 m^2^–25,200 m^2^	Modern Infrastructure (Offices and Commercial Spaces)

**Table 11:** Comparison of positioning accuracy in BLE-based systems using RSSI and AoA techniques.

Technique	Studies	Error Range (m)	Experimental Area (m^2^)	Environmental Context
RSSI	41	0.00023 m–6.01 m	8.24 m^2^–4240 m^2^	Diverse Applications (Single Rooms to Office Floors)
AoA	5	0.0032 m–1.67 m	10 m^2^–63 m^2^	Small, Controlled Environments (Primarily Labs)

**Table 12:** Comparative summary of ML approaches for the positioning stage in RSSI-based systems.

Aspect	Wi-Fi RSSI Systems	BLE RSSI Systems
Dominant Algorithms	KNN serves as a strong baseline, but there is a clear	Dominated by lightweight algorithms,
	and diverse adoption of DL (CNNs, LSTMs,	primarily KNN and its variants (WKNN, IKNN).
	DNNs).	
Algorithmic Diversity	High. Includes a wide range of supervised, ensemble	Low. Exploration of DL models for positioning is limited
	(RF, XGBoost), and advanced DL models	and largely in an early, exploratory stage. The focus
	(Transformers, GANs).	remains on efficiency.
Primary Challenges	Signal instability in dynamic environments, device	High signal volatility and lower data granularity, which
	heterogeneity, and the high cost of radio map	complicates feature extraction. Energy efficiency is a key
	maintenance.	design constraint.
Key Research Trend	A clear shift towards end-to-end DL models and	Balancing performance with energy efficiency. The focus
	domain adaptation to improve generalization and reduce	remains on optimizing classical positioning algorithms.
	calibration dependency.	

## Data Availability

No new data were created or analyzed in this study.

## Supplementary Materials

The following supporting information can be downloaded at: https://www.mdpi.com/article/10.3390/s25226946/s1, Table S1: PRISMA 2020 Checklist.

## Abbreviations

The following abbreviations are used in this manuscript:

**AoA**	Angle of Arrival
**AP**	Access Point
**BLE**	Bluetooth Low Energy
**CNN**	Convolutional Neural Network
**CSI**	Channel State Information
**DL**	Deep Learning
**DNN**	Deep Neural Network
**DoA**	Direction of Arrival
**DT**	Decision Tree
**EC**	Exclusion Criteria
**FFNN**	Feed-Forward Neural Network
**FTM**	Fine Timing Measurement
**GA**	Genetic Algorithms
**GDPR**	General Data Protection Regulation
**GMM**	Gaussian Mixture Model
**GPS**	Global Positioning System
**GPR**	Gaussian Process Regression
**HAR**	Human Activity Recognition
**HMM**	Hidden Markov Model
**IC**	Inclusion Criteria
**IKNN**	Improved K-Nearest Neighbour
**IMU**	Inertial Measurement Unit
**IoT**	Internet of Things
**IPS**	Indoor Positioning System
**KNN**	K-Nearest Neighbour
**LOS**	Line-of-Sight
**LSTM**	Long Short-Term Memory
**ML**	Machine Learning
**MLP**	Multilayer Perceptron
**MRQ**	Main Research Question
**NB**	Naive Bayes
**NLOS**	Non Line-of-Sight
**PCA**	Principal Component Analysis
**PRISMA**	Preferred Reporting Items for Systematic Reviews and Meta-Analyse
**PSO**	Particle Swarm Optimization
**RF**	Random Forest
**RF**	Radio Frequency
**RFID**	Radio Frequency Identification
**RNN**	Recurrent Neural Network
**RQ**	Research Question
**RSSI**	Received Signal Strength Indicator
**RTT**	Round Trip Time
**SLR**	Systematic Literature Review
**SVM**	Supported Vector Machine
**SVR**	Support Vector Regression
**ToA**	Time of Arrival
**ToF**	Time of Flight
**UWB**	Ultra-Wideband
**VLC**	Visible Light Communication
**VLP**	Visible Light Positioning
**Wi-Fi**	IEEE 802.11Wireless LAN
**WKNN**	Weighted K-Nearest Neighbour
